# Peimine, an Anti-Inflammatory Compound from Chinese Herbal Extracts, Modulates Muscle-Type Nicotinic Receptors

**DOI:** 10.3390/ijms222011287

**Published:** 2021-10-19

**Authors:** Armando Alberola-Die, José Antonio Encinar, Raúl Cobo, Gregorio Fernández-Ballester, José Manuel González-Ros, Isabel Ivorra, Andrés Morales

**Affiliations:** 1 División de Fisiología, Departamento de Fisiología, Genética y Microbiología, Universidad de Alicante, Apdo. 99, E-03080 Alicante, Spain; alberoladie.armando@ua.es (A.A.-D.); raulcobo22@gmail.com (R.C.); isabel.ivorra@ua.es (I.I.); 2 Instituto de Investigación, Desarrollo e Innovación en Biotecnología Sanitaria de Elche (IDiBE), Universidad Miguel Hernández, E-03202 Elche, Spain; jant.encinar@umh.es (J.A.E.); gregorio@umh.es (G.F.-B.); gonzalez.ros@umh.es (J.M.G.-R.)

**Keywords:** peimine, traditional Chinese medicine, anti-inflammatory compound, nicotinic receptors, electrophysiological recordings, *Xenopus* oocytes, molecular docking, dynamics simulations

## Abstract

*Fritillaria* bulbs are used in Traditional Chinese Medicine to treat several illnesses. Peimine (Pm), an anti-inflammatory compound from *Fritillaria*, is known to inhibit some voltage-dependent ion channels and muscarinic receptors, but its interaction with ligand-gated ion channels remains unexplored. We have studied if Pm affects nicotinic acetylcholine receptors (nAChRs), since they play broad functional roles, both in the nervous system and non-neuronal tissues. Muscle-type nAChRs were incorporated to *Xenopus* oocytes and the action of Pm on the membrane currents elicited by ACh (*I_ACh_*s) was assessed. Functional studies were combined with virtual docking and molecular dynamics assays. Co-application of ACh and Pm reversibly blocked *I_ACh_*, with an IC_50_ in the low micromolar range. Pm inhibited nAChR by: (i) open-channel blockade, evidenced by the voltage-dependent inhibition of *I_Ach_*, (ii) enhancement of nAChR desensitization, revealed by both an accelerated *I_ACh_* decay and a decelerated *I_ACh_* deactivation, and (iii) resting-nAChR blockade, deduced from the *I_ACh_* inhibition elicited by Pm when applied before ACh superfusion. In good concordance, virtual docking and molecular dynamics assays demonstrated that Pm binds to different sites at the nAChR, mostly at the transmembrane domain. Thus, Pm from *Fritillaria* bulbs, considered therapeutic herbs, targets nAChRs with high affinity, which might account for its anti-inflammatory actions.

## 1. Introduction

The scientific interest in Traditional Chinese medicines (TCMs) has bloomed in the last decades, as they provide a broad source of compounds of putative clinical relevance. The active ingredients of TCM plants include: (i) alkaloids, such as peimine (Pm), found in *Fritillaria* bulbs (*F*b), which are commonly used to treat cough and asthma [1,2], (ii) terpenoids, as ginsenoides from *Panax ginseng*, which might reverse multidrug resistance of certain chemotherapeutic drugs [3], (iii) phenols and flavonoids (polyphenolic compounds), which are common in medicinal plants and own strong antioxidant and anti-inflammatory activities [4], tannic acid-related gallotannins and polyphenols, which are relatively abundant in green tea, inhibit TMEM16A, a calcium-activated chloride channel [5] and some flavonoids, as quercetin or genistein, act as positive allosteric modulators of α7 nicotinic receptors [6], and (iv) other compounds, as cinnamaldehyde, obtained from cinnamon cortex, which is known to activate TMEM16A [7].

Pm, also known as verticine, has been related to diverse therapeutic actions [8], including: (i) anti-inflammatory and analgesic, (ii) antitumor, inhibiting proliferation of human leukemia or lung cancer, (iii) expectorant, (iv) sedative, besides its analgesic action, (v) antihypertensive, (vi) a blocker of voltage-dependent ion channels, including both Na^+^ and K^+^ channels, and (vii) antimuscarinic, mainly acting on the M2 receptor subtype. Of note, Pm has a very low oral bioavailability, mainly because of its limited water solubility, and its intestinal absorption seems to take place through active transport, being pH-dependent [9]. In fact, by using Caco-2 cell monolayers, it has been estimated that the percentage of Pm absorbed would not exceed 2%, which means a poor absorption [9]. Consequently, it is expected that Pm actions should be elicited by high-affinity binding to specific targets, likely involved in inflammation, pain, smooth-muscle relaxation, sedation, or exocrine secretion. To date, the main effects of Pm have been attributed to different mechanisms of action, including: (i) use-dependent inhibition of voltage-dependent Nav1.7 channels, which would promote pain relief [10]; (ii) inhibition of voltage-dependent K^+^ channels, including the Kv1.3 expressed in lymphocytes and other non-excitable cells; in fact, Kv1.3. blockade by Pm has been related to its anti-inflammatory action [10]. Furthermore, Pm inhibits hERG (human ether-a-go-go related gene) channels by promoting their inactivation [11]. Remarkably, hERG channels play a critical role in the repolarization of myocardial cells and, thus, in the cardiac excitability; (iii) M2 muscarinic receptor inhibition, which might account for asthma amelioration, since M2 receptors are involved in airway smooth-muscle contraction [12]; (iv) angiotensin-converting enzyme inhibition, which would contribute to its antihypertensive action [13]; (v) enhancement of intracellular Ca^2+^ concentration, promoting phosphorilation of both Ca^2+^/calmodulin-dependent protein kinase II (CaMKII) and c-Jun N-terminal kinase (JNK), which inhibits growth and motility of cancer cells [14]; (vi) inhibition of P-glycoprotein expression in drug-resistance cells, reducing the ability of cancer cells to survive from chemotherapy [3]. At present, *F*b are regarded as therapeutic herbs, despite that one of its main active compounds, Pm, blocks hERG channels (IC_50_ circa 40 µM) and, hence, it might trigger severe alterations in cardiac excitability. The fact that *Fritillaria* intake is yet considered safe, strongly suggests that its bioactive compounds, as Pm, must bind to specific targets with affinities much higher than those required to mediate the inactivation of hERG channels. However, presently, most putative therapeutic actions of Pm are elicited at concentrations similar to, or above, those required to block hERG channels, which suggests that Pm should act on additional molecular targets. Interestingly, as far as we know, it has not been yet assessed if Pm interacts with ligand-gated ion channels. This is rather remarkable since, for instance, nicotinic acetylcholine (ACh) receptors (nAChRs) are widely expressed in non-neuronal cells, including macrophages [15], and different subtypes of nAChRs have been involved in pain, inflammation, lung cancer, and proliferation of smooth-muscle and endothelial cells [15,16].

The nAChR belongs to the “Cys-loop” family of receptors, which are involved in fast synaptic transmission. Although all nAChRs are pentameric proteins, there is a large heterogeneity in their structures. Thus, whereas some nAChRs are homomeric, as those constituted by α7-10 subunits, others are heteromeric, containing specific combinations of α1-6, β1-4, γ, ε, and δ subunits. This large assortment of structural conformations of nAChRs accounts for a huge heterogeneity of functional and pharmacological properties of nAChRs. To date, the best characterized nAChR is the muscle type, located at postsynaptic membranes of both skeletal muscle fibers and electrocytes of some electric fishes, such as *Torpedo*. This nAChR is composed of 2α1, 1β1, 1δ, and either 1ε (adult-type) or 1γ (fetal-type) subunits, disposed delimiting a central channel pore [17,18,19,20].

This work aimed, first, to study the effects of Pm on nAChRs, since these interactions might account for some of its therapeutic actions, second, to decipher the mechanisms by which Pm modulate the activity of nAChR, and third, to predict, by docking and molecular dynamics (MD) assays, the putative sites at which Pm binds to the nAChR, in either open or closed conformations, to mediate its actions. Our results indicate that Pm is a powerful modulator of nAChR function, acting at the submicromolar range. Actually, Pm binds to the nAChR with high affinity at multiple sites, mostly, but not exclusively, located at the transmembrane domain (TMD), and inhibits its function by different mechanisms. Correlation of structural data, concerning the specific binding sites of Pm at the nAChR, with the functional effects elicited by this molecule should contribute to expand our understanding of the modulation of nAChRs by molecules of therapeutic relevance.

## 2. Results

### 2.1. I_ACh_ Blockade by Pm

**Figure 1:**
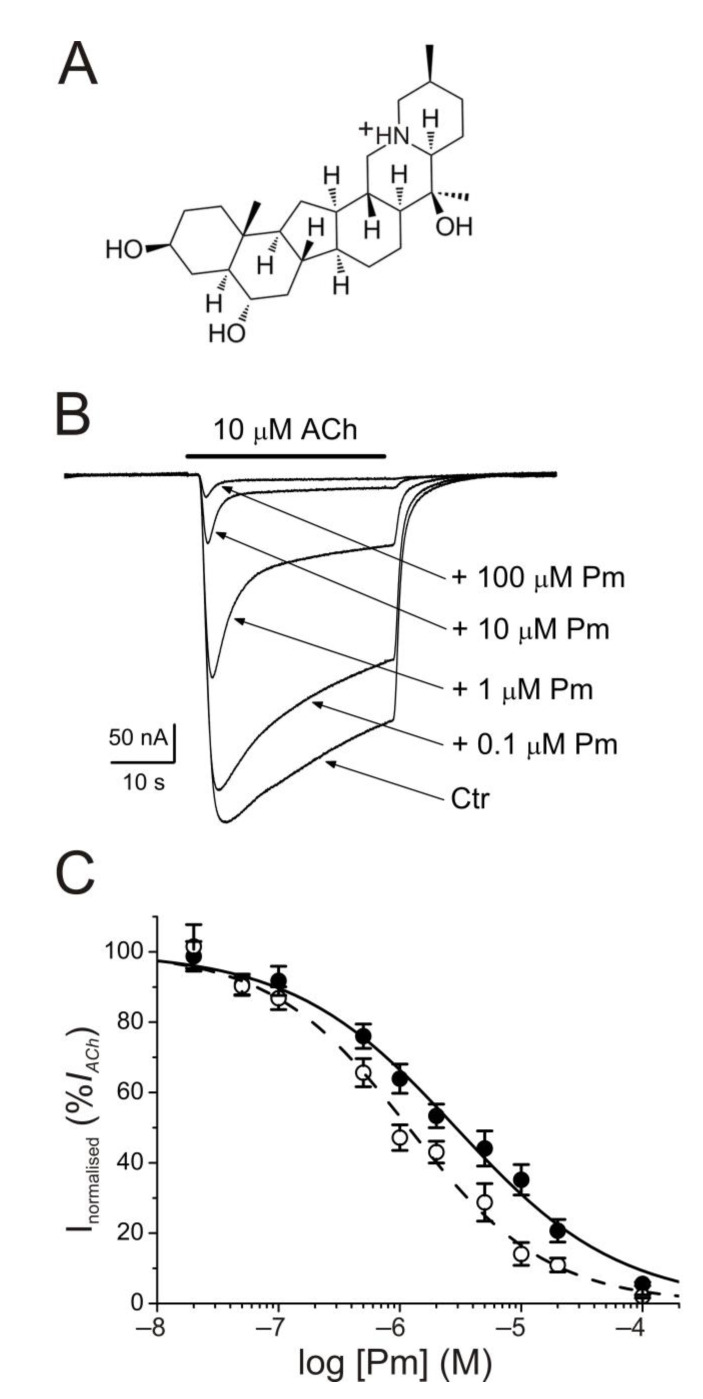
Inhibition of acetylcholine-elicited currents (*I_ACh_s*) by peimine (Pm). (**A**) Molecular structure of Pm showing the charged nitrogen. (**B**) Superimposed *I_ACh_s* evoked by 10 µM ACh either alone (Ctr) or co-applied with different Pm concentrations, as stated on the right. Note that Pm accelerates *I_ACh_* decay when applied at concentrations of 0.1 μM or above. Hereafter, unless otherwise stated, the holding potential was −60 mV, downward deflections represent inward currents and the bars above recordings indicate the timing of drug application. (**C**) Pm concentration-*I_ACh_* inhibition relationship. *I_ACh_* amplitudes at their peak (*I_p_*; filled symbols) and at their steady state (*I_ss_*, measured 20 s after the peak; open symbols) were normalized to the *I_ACh_* evoked by ACh alone and plotted against the logarithm of Pm concentration. Solid and dashed lines are sigmoid curves fitted to *I_p_* and *I_ss_* data, respectively. Error bars indicate SEM. Each point is the average of 5–16 oocytes from 3–8 frogs.

The membrane conductance of oocytes either uninjected or bearing microtransplanted nAChRs was unaffected by bathing the cell with Pm (up to 100 µM) while holding the membrane potential at −60 mV. By contrast, co-application of ACh (10 µM) together with Pm (0.02–100 µM) to microinjected oocytes reversibly reduced the peak-amplitude (*I_p_*) of *I_ACh_*, in a dose-dependent manner (Figure 1B), following a sigmoid function (Figure 1C). At Pm concentrations over 0.1 µM, the extent of *I_ACh_* inhibition measured 20 s after *I_p_* (*I_ss_*) was greater than that corresponding to *I_p_* values. In this way, the IC_50_ and n_H_ values (see equation 1) for the *I_p_* were 2.9 µM (confidence interval (CI), 2.0–4.3 µM; *n* = 5–16, *N* = 3–8) and 0.7 ± 0.1, respectively, whereas the dose-inhibition curve for the *I_ss_* displayed a lower IC_50_ (1.2 µM; CI 0.9–1.5 µM) but a similar slope (0.8 ± 0.1; the same cells and donor frogs as above; Figure 1C). Most likely, this lower IC_50_ for *I_ss_* is because of the enhancement of nAChR desensitization by Pm (see below).

The specificity of Pm effects on muscle-type nAChR blockade was assessed by testing its effects on GABA subtype A receptors (GABA_A_Rs), which belong to the same Cys-loop family. For these experiments, oocytes were microinjected with rat brain synaptosomal membranes, which allowed the incorporation of GABA_A_Rs into the oocyte membrane [21]. These cells were later challenged with GABA (1 mM) either alone or together with Pm (up to 100 µM). Both *I_GABA_* amplitude and kinetics were rather unaffected by the presence of Pm (Supplementary Figure S1), in contrast to the marked inhibition of muscle-type nAChRs by Pm.

### 2.2. Competition Assays

**Figure 2:**
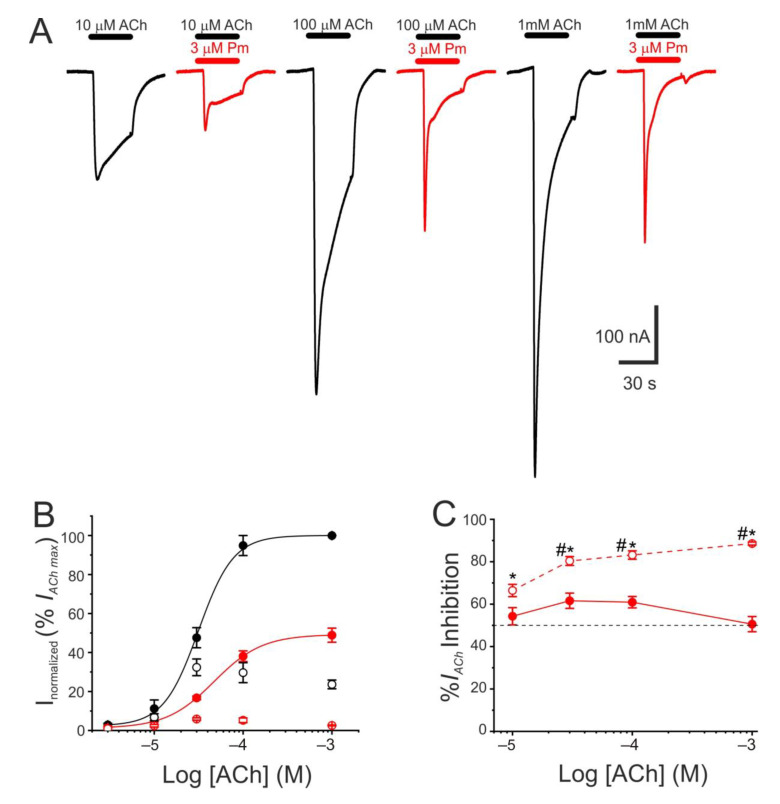
Pharmacological profile of nAChR blockade by Pm. (**A**) Representative *I_ACh_s* evoked by ACh at 10 µM (left), 100 µM (middle), or 1 mM (right), either alone (black recordings) or together with 3 µM Pm (red recordings). (**B**) ACh concentration-*I_ACh_* amplitude relationship when the cell was bathed with either just ACh (black color; filled symbols denote *I_p_* values whereas open circles correspond to *I_ss_*) or ACh plus 3 µM Pm (red symbols). The EC_50_ values of the sigmoid curves fitting the experimental data were 31 µM (range 11–52 µM) and 47 µM (range 46–49 µM) for control and 3 µM Pm, respectively. (**C**) Percentage of *I_p_* (solid symbols) and *I_ss_* (open symbols) inhibition elicited by 3 µM Pm when co-applied with the indicated ACh concentrations. (*) indicates significant differences between *I_p_* and *I_ss_* inhibition, for each ACh concentration (*p* < 0.05, paired *t*-test). (#) indicates significant differences among *I_ss_* inhibition at 10 µM ACh and other concentrations (*p* < 0.05, ANOVA followed by Bonferroni *t*-test). Each point of panels B and C is the average of 4–14 cells from 1–2 donors.

The pharmacological profile of nAChR inhibition by Pm was determined by superfusing oocytes with ACh at different concentrations (3, 10, 30, 100 µM, and 1 mM) either alone or together with Pm at a concentration close to its IC_50_ (3 µM). Co-application of 10 µM ACh with 3 µM Pm halved *I_p_*, as expected from the computed IC_50_ (Figure 1C). Similar percentages of *I_p_* inhibition were found when Pm was co-applied with different ACh concentrations (Figure 2A–C), suggesting that Pm is acting as a non-competitive blocker. Notably, the percentages of *I_ss_* inhibition attained by co-applying Pm (3 µM) with different ACh concentrations were higher than those corresponding to the *I_p_* inhibition (Figure 2A,C). Furthermore, the percentage of *I_ss_* inhibition increased significantly as the ACh concentration augmented (Figure 2C). This ACh concentration-dependence of *I_ss_* inhibition might be related to the enhancement of nAChR desensitization by Pm (see below), since the rate of desensitization is known to be dependent on ACh concentration [22,23].

### 2.3. Enhancement of nAChR Desensitization by Pm

**Figure 3:**
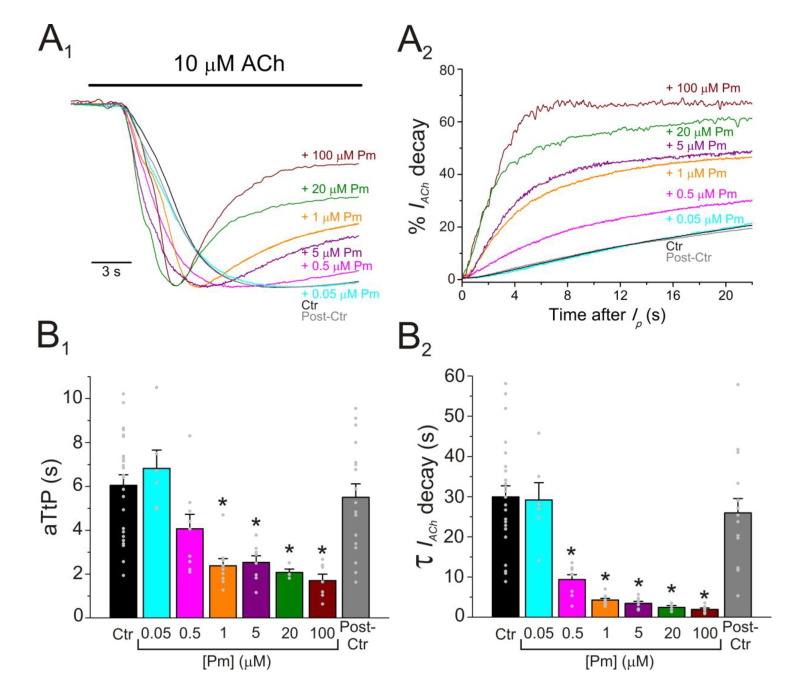
Pm accelerates *I_ACh_* decay and shortens the time to reach *I_p_.* (**A_1_,A_2_**) Superimposed *I_ACh_s* elicited by 10 µM ACh either alone (black and grey recordings) or together with different Pm concentrations (shown at right). *I_ACh_*s were scaled to the same *I_p_* amplitude to better compare the differences in time to reach *I_p_* (aTtP; **A_1_**) and kinetics of *I_ACh_* decay after *I_p_* (**A_2_**). (**B_1_**,**B_2_**) Column graphs displaying Pm effects on aTtP (**B_1_**) and τ-values of *I_ACh_-*decay (**B_2_**). (*) indicates significant differences among *I_ACh_*s in presence of Pm (colored columns; same color code as in (**A_1_**,**A_2_**)) and their control values (Ctr, black column; *p* < 0.05, ANOVA and Bonferroni *t*-test). Note that post-control values (after Pm applications; grey column) were similar to control ones. Each point is the average of 4–24 cells (*N* = 3–12).

As previously demonstrated, co-application of ACh with Pm at concentrations over 0.1 µM accelerated *I_ACh_* decay (Figure 1B), which, in turn, resulted in a large percentage of *I_ss_* inhibition, as compared to *I_p_* (Figure 1B,C). This acceleration of *I_ACh_* decay by Pm might be due to: (i) slow *I_ACh_* blockade, (ii) enhancement of nAChR desensitization, and (iii) a combination of both. Aiming to unravel the involvement of each one of these options in boosting *I_ACh_* decay we conducted several complementary experimental protocols. First, ACh (10 µM) was co-applied with different Pm concentrations (0.05–100 µM) and both the time to reach *I_p_* (aTtP) and the *I_ACh_* decay kinetics were determined (Figure 3).

Acceleration of *I_ACh_* decay was dependent on Pm concentration and significantly increased at concentrations over 0.5 µM Pm (Figure 3A_1_,A_2_,B_2_). Actually, the *I_ACh_* decay time-constant (τ) decreased from roughly 30 s for control *I_ACh_*s to less than 2 s when ACh and 100 µM Pm were co-applied (Figure 3B_2_). Furthermore, Pm elicited changes in the aTtP (Figure 3B_1_), which paralleled fairly well the acceleration of *I_ACh_* decay (Figure 3B_2_). Noticeably, a shortened aTtP has been previously related to enhancement of nAChR desensitization, as reported for the action of both lidocaine and 2,6-dimethylaniline (DMA) on this receptor [24,25].

**Figure 4:**
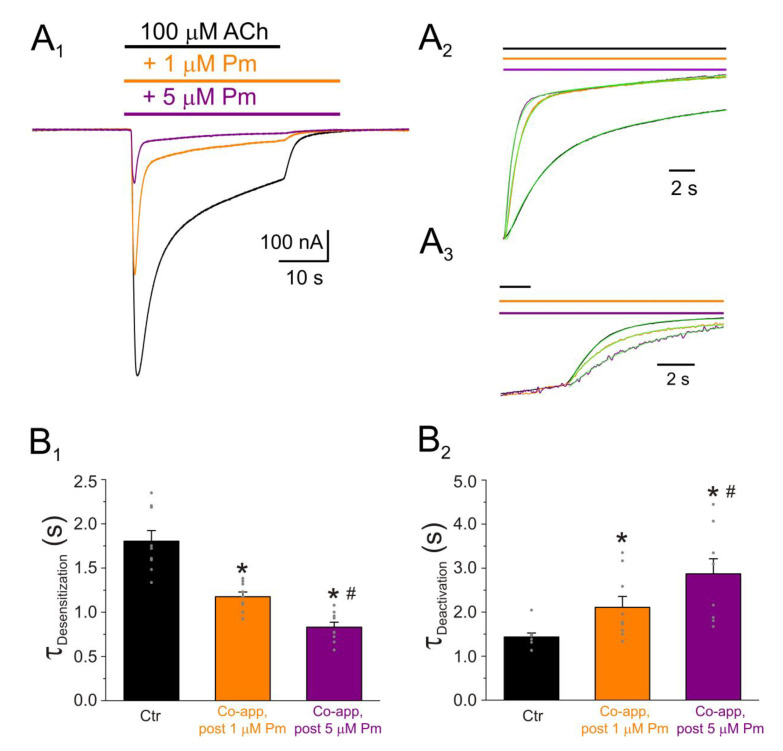
*I_ACh_* decay and deactivation kinetics depend on Pm concentration. (**A_1_**,**A_2_**,**A_3_**) Representative *I_ACh_*s elicited by 100 µM ACh either alone (black recording) or together with 1 (orange) or 5 (purple) µM Pm (**A_1_**). Pm superfusion remained 12 s after ACh washout (as indicated by the application bars). These recordings were normalized to either the same *I_p_*, to better compare their *I_ACh_* decay (**A_2_**), or the same *I_ss_*, to facilitate comparisons of deactivation kinetics (**A_3_**). (**B_1_**,**B_2_**) Column bar plots displaying the effect of 1 (orange) or 5 µM (purple) Pm on the *I_ACh_* decay time-constant (τ_Desensitization_; (**B_1_**)) and the deactivation kinetics (τ_Deactivation_; (**B_2_**)), as compared to control *I_ACh_*s (in the presence of ACh alone; black). (*) indicates significant differences with the control group (*p* < 0.05, paired *t*-test) and (#) indicates differences between 1 and 5 µM Pm groups (*n* = 9, *N* = 3; the same cells for all comparisons; *p* < 0.05, paired *t*-test). Notice that Pm accelerated the desensitization rate and slowed down the deactivation kinetics.

Moreover, the kinetics of *I_ACh_* tails (deactivation) differed depending on the presence or absence of Pm while rinsing ACh out. In these experiments, 100 µM ACh, which elicits large nAChR desensitization, was bathed alone or together with either 1 or 5 µM Pm for 32 s. Afterwards, ACh was removed, keeping the cell superfused with normal Ringer with atropine (ANR) either alone or together with 1 or 5 µM Pm (Figure 4). As previously demonstrated, 1 µM Pm accelerated *I_ACh_* decay and this effect was more pronounced when the cell was bathed with 5 µM Pm (Figure 4A_1_,A_2_,B_1_). Thus, the ratio of *I_ACh_* decay time constant values in the presence of 1 or 5 µM Pm versus those in ACh alone were significantly smaller than 1 (0.67 ± 0.04 and 0.48 ± 0.05, respectively; same cells in both groups; *n* = 9, *N* = 3; *p* < 0.001, one-sample *t*-test). Furthermore, 5 µM Pm elicited a greater nAChR blockade and faster *I_ACh_* decay as compared to 1 µM Pm (1.18 ± 0.05 s against 0.83 ± 0.06 s for *I_ACh_* decay at 1 and 5 µM Pm, respectively; *p* = 0.002, paired *t*-test, same cells as above). As previously reported [26], deactivation of control *I_ACh_*s, elicited by ACh washout, followed an exponential function with a time course of roughly 1.5 s (this value likely limited by the solution exchange kinetics [26]).

Noticeably, the presence of Pm in the ANR decelerated *I_ACh_* deactivation in a dose-dependent manner (τ_Deactivation_ of 2.1 ± 0.2 s and 2.9 ± 0.3 s for *I_ACh_*s in the presence of 1 µM and 5 µM Pm, respectively; *p* = 0.044, same cells as above; Figure 4B_2_). In fact, the ratios of τ_Deactivation_ of *I_ACh_*s in the presence and the absence of Pm were 148 ± 17% and 206 ± 30%, for 1 and 5 µM Pm, respectively. These later percentages most likely are underestimated since the actual ratios are conditioned by the apparent kinetics of control *I_ACh_* deactivation. Remarkably, if Pm actually enhances nAChR desensitization, a deceleration of *I_ACh_* deactivation is expected, since desensitized nAChRs display a higher affinity for ACh [22,27].

### 2.4. nAChRs Blockade by Pm Is Voltage-Dependent

**Figure 5:**
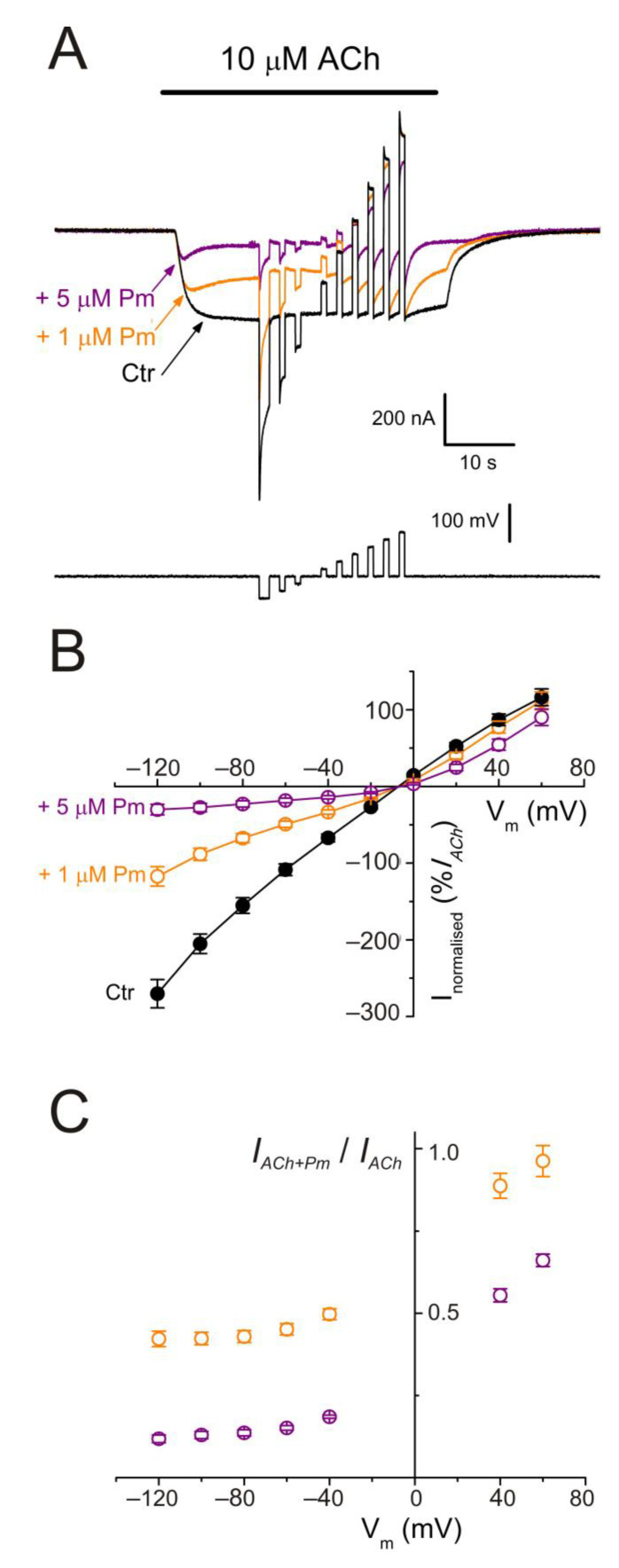
nAChR blockade by Pm is voltage-dependent. (**A**) *I_ACh_*s evoked by 10 µM ACh alone (black recording) or in the presence of either 1 (orange) or 5 µM (purple) Pm when applying voltage pulses from −120 to +60 mV, as illustrated underneath. (**B**) Net *i/v* relationship of *I_ACh_*s elicited by the protocol shown in (**A**). Black symbols are for control *I_ACh_*s, whereas those evoked in the presence of Pm are drawn in either orange (+1 µM Pm) or purple (+5 µM Pm). Net *I_ACh_*s were normalized as the percentage of their control *I_ACh_* at −60 mV (*n* = 5–11; *N* = 2–3). (**C**) Plot displaying the *I_ACh_* remnant after co-application of either 1 (orange) or 5 µM (purple) Pm (*I_ACh+Pm_*), normalized to their control (*I_ACh_*), versus the membrane potential (same cells as in (**B**)). Notice that 1 µM Pm, in contrast to 5 µM, did not significantly decrease *I_ACh_* at +60 mV.

Pm contains a protonable amine group (see Figure 1A), being its strongest basic pK_a_ 10.56 (data from Chemicalize, https://chemicalize.com/; accessed on 30 March 2017). Consequently, more than 99.9% of Pm molecules are in a charged form at the recording pH. To unravel if *I_ACh_* inhibition exerted by Pm is voltage-dependent, voltage jumps (from −120 to +60 mV, in 20 mV steps) were imposed to oocytes superfused with ANR or during the *I_ACh_* plateau elicited by 10 µM ACh, either alone or co-applied with 1 or 5 µM Pm (Figure 5A).

The *i/v* curves of net *I_ACh_*s elicited in the presence of Pm demonstrated that nAChR blockade by Pm was voltage-dependent, the more hyperpolarized the cell membrane, the larger the *I_ACh_* blockade (Figure 5). Furthermore, *i/v* curves displayed a reversal potential close to −5 mV, indicating that channel-permeability properties were unaffected by the presence of Pm. Noteworthy, 1 µM Pm only inhibited *I_ACh_* at negative potentials, suggesting that Pm is electrostatically blocking the channel pore and, thus, causing an open-channel blockade. The relationship between the percentages of *I_ACh_* remnant and voltage displayed a slight slope at negative potentials (Figure 5C), suggesting that Pm binds into the channel pore at a shallow site from the extracellular side. The increase of Pm concentration to 5 µM enhanced *I_ACh_* inhibition and the blockade was evident both at positive and negative potentials (Figure 5B,C). Thus, at +60 mV, the *I_ACh_* remnant in the presence of 5 µM Pm was 0.66 ± 0.02, *n* = 5, *N* = 2 (*p* < 0.05, one sample *t*-test) whereas *I_ACh_* was unaffected by 1 µM Pm (*I_ACh_* remnant 0.96 ± 0.05, *n* = 11, *N* = 3; *p* > 0.05, one sample *t*-test). Therefore, Pm, at 5 µM, should bind to additional sites beyond those involved in open-channel blockade. 

### 2.5. Kinetics of nAChR Blockade by Pm and Its Recovery

**Figure 6:**
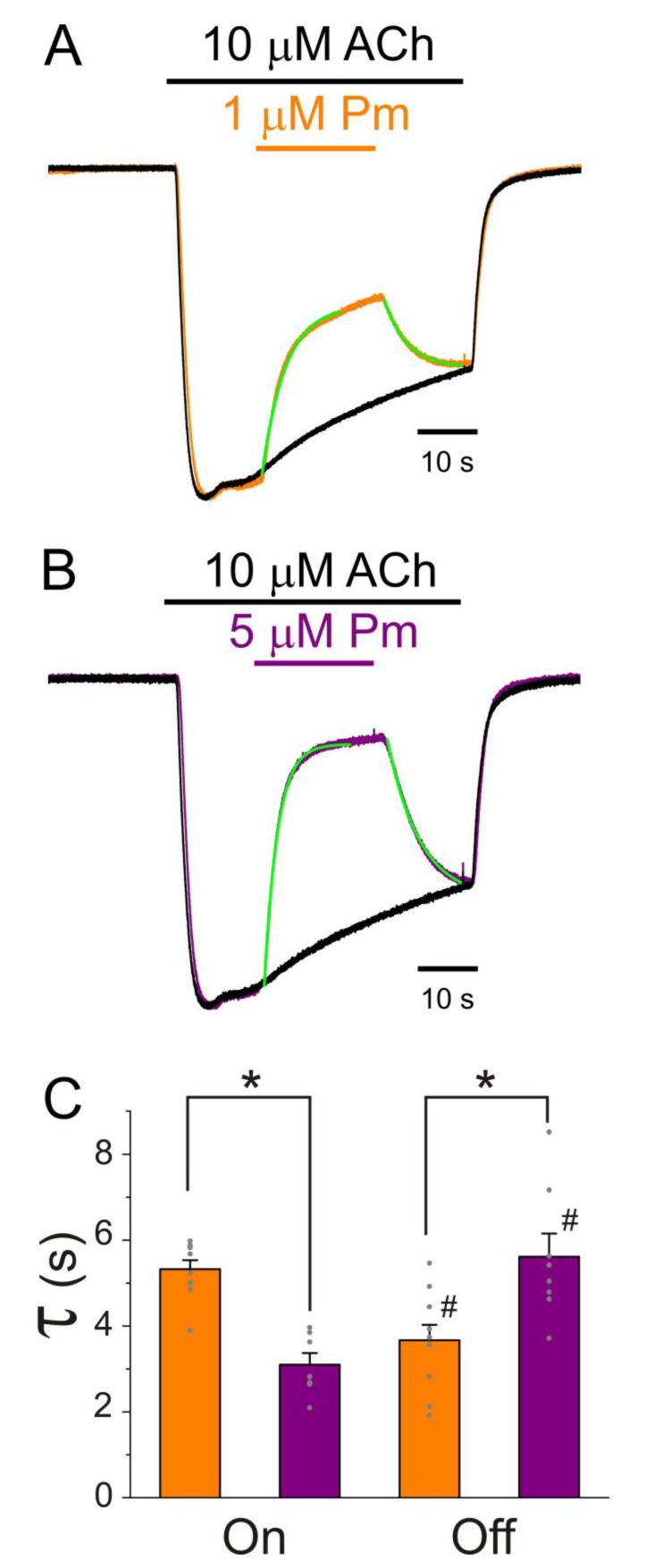
Blockade of open nAChRs by Pm. (**A**,**B**) Superimposed *I_ACh_s* elicited by 50 s pulses of 10 µM ACh either alone (black recordings) or together with 1 (**A**) or 5 µM (**B**) Pm, applied at the *I_ACh_* plateau. *I_p_*s were normalized to the same amplitude to facilitate the kinetics comparisons. The kinetics of *I_ACh_* inhibition and its recovery from blockade followed exponential functions (green traces (**A**,**B**)). (**C**) Column graph of the τ values found for *I_ACh_* blockade onset (“On” columns) when 1 (orange) or 5 µM (purple) Pm was co-applied with 10 µM ACh. The “Off” columns correspond to the kinetics of recovery (τ) from blockade, following Pm removal (same color code). (*) indicates significant differences of τ values between Pm concentrations for either “On” or “Off” data (*p* < 0.05, ANOVA and Bonferroni *t*-test). (#) denotes differences between “On” and “Off” values for either 1 or 5 µM Pm (*p* < 0.05, paired *t*-test). Data are for 10 and 8 oocytes (*N* = 5) for 1 µM and 5 µM Pm, respectively.

To better characterize the open-channel blockade of nAChRs by Pm, we tested the effect of a 20 s pulse of either 1 or 5 µM Pm applied during the *I_ACh_* plateau elicited by a 50 s pulse of 10 µM ACh (Figure 6A,B). Pm addition to the bathing solution evoked a marked *I_ACh_* inhibition, decreasing 42 ± 2% (*n* = 10, *N* = 5) and 71 ± 1% (*n* = 8, *N* = 5) by 1 and 5 µM Pm, respectively (*p* < 0.05, *t*-test). The τ values for the fast blockade phase, estimated by fitting a single exponential function to the recordings, were 5.3 ± 0.2 and 3.1 ± 0.3 s for 1 and 5 µM Pm, respectively (same cells as above; *p* < 0.05, *t*-test; Figure 6C).

**Figure 7:**
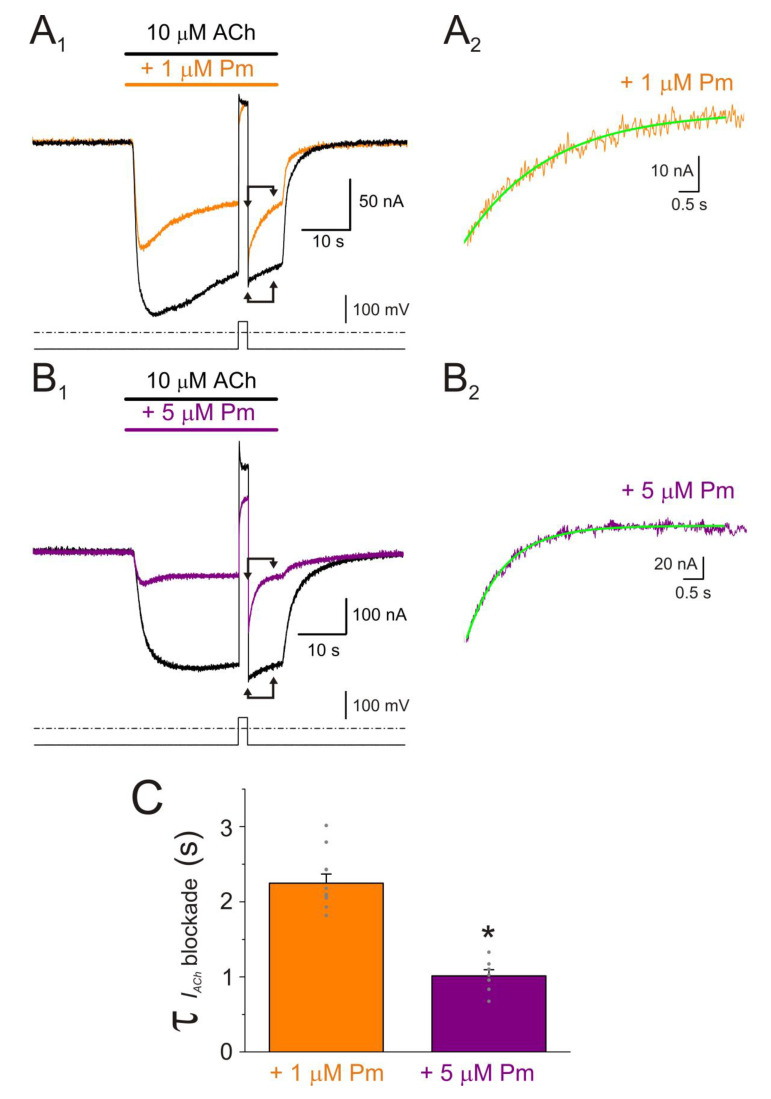
Kinetics of the voltage-dependent blockade of nAChR by Pm. (**A_1_**,**B_1_**) *I_ACh_*s elicited by 10 µM ACh either alone (black recordings) or in the presence of 1 (orange; (**A_1_**)) or 5 µM (purple; (**B_1_**)) Pm, at −60 mV. A 2 s voltage jump to +40 mV was given at the *I_ACh_* plateau to unplug the channel pore of the positively-charged Pm. Membrane leak-currents (recorded in the absence of ACh) have been subtracted. (**A_2_**,**B_2_**) Zooming in to the area indicated by the arrows in panels (**A_1_**,**B_1_**) (just after the voltage jump). The τ of the voltage-dependent blockade of nAChRs by Pm was determined by fitting an exponential function (green curve over the recording) to the net *I_ACh_* decay. Before fitting, the smaller and slower *I_ACh_*s evoked by ACh alone (black recordings of panels (**A_1_**,**B_1_**)) were subtracted from the *I_ACh_*s in the presence of Pm. (**C**) Column-graph of τ values of the voltage-dependent blockade of nAChR by 1 and 5 µM Pm (same color code as in panels (**A**,**B**)). (*) indicates significant differences of τ values between both Pm concentrations (*p* < 0.05, *t*-test). Data are for 10 (*N* = 3) and 7 (*N* = 2) oocytes for 1 µM and 5 µM Pm, respectively.

These τ values were rather slow, as compared to those previously reported, under similar experimental conditions, for *I_ss_* inhibition elicited by different local anesthetics (LAs), as tetracaine or benzocaine, at their IC_50_. In fact, for these latter compounds, τ values were faster than the solution exchange kinetics (roughly 1.5 s [26,28]). Furthermore, the voltage-dependent blockade of nAChRs by Pm allowed us to determine more accurately the rate of open-channel blockade, by jumping the membrane potential during the *I_ACh_* elicited in the presence of Pm. Thus, during the *I_ACh_* plateau elicited, at −60 mV, by 10 µM ACh either alone or in the presence of 1 or 5 µM Pm, the membrane potential was stepped to +40 mV for 2 s, to remove the open-channel blockade elicited by Pm. Following this pulse, the membrane potential returned to −60 mV, to determine the kinetics of the open-channel blockade by Pm (Figure 7A_1_,B_1_). The *I_ACh_* blockade by Pm followed an exponential function (Figure 7A_2_,B_2_) with τ values of 2.25 ± 0.12 s (*n* = 10, *N* = 3) and 1.01 ± 0.08 s (*n* = 7; *N* = 2) for 1 and 5 µM Pm, respectively. As expected, the kinetics of the voltage-dependent blockade of *I_ACh_* accelerated by increasing Pm concentration (*p* < 0.05, *t*-test; Figure 7C). These τ values were shorter than those obtained by direct Pm superfusion during the *I_ACh_* plateau (Figure 6), most likely because they were not affected by the kinetics of solution exchange.

The recovery of nAChR from Pm blockade was also rather slow, in the range of several seconds, and followed a single exponential function (Figure 6A–C). However, in contrast to the development of nAChR blockade, the recovery decelerated by raising Pm concentration (Figure 6C).

### 2.6. Effect of Pm-Application Timing and Holding Potential on nAChR Blockade

**Figure 8:**
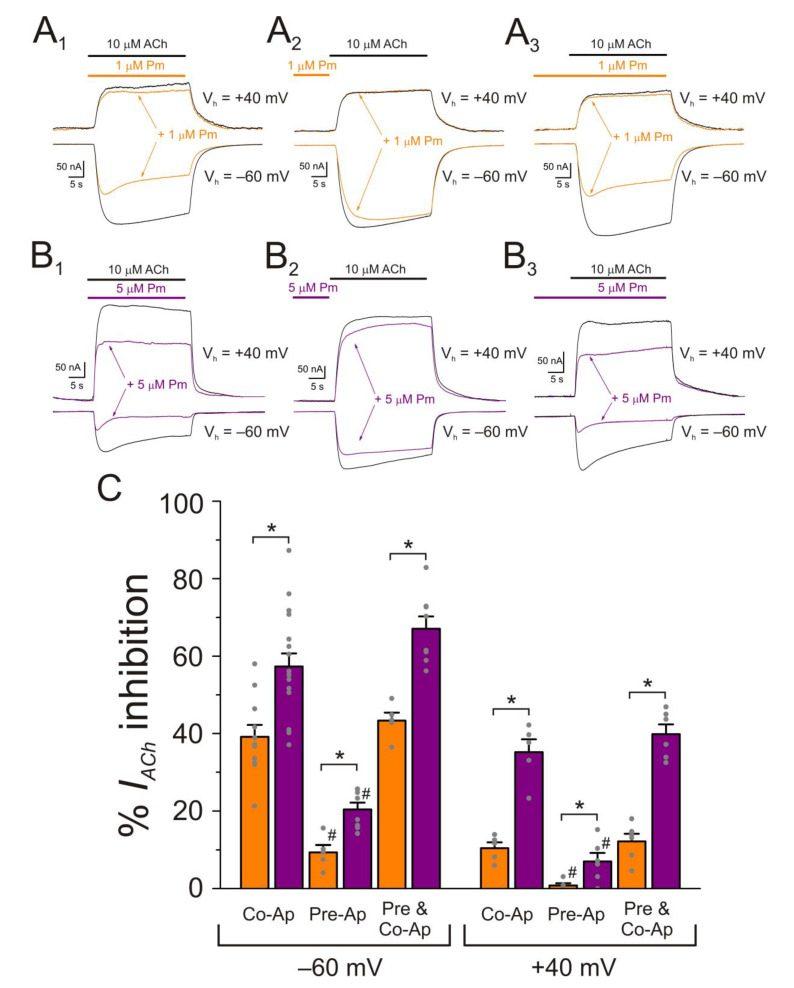
Effect of Pm-application timing and holding potential on nAChR blockade. (**A_1_**–**A_3_**) *I_ACh_s* elicited at −60 mV (downward deflections) and at +40 mV (upward deflections) by co-application of 10 µM ACh and 1 µM Pm (**A_1_**), solely Pm pre-application before superfusing the agonist (**A_2_**) or Pm pre-application followed by its co-application with ACh (**A_3_**). (**B_1_**–**B_3_**) As in panels (**A_1_**–**A_3_**), but in the presence of 5 µM Pm instead of 1 µM. (**C**) Column graph shows the percentages of *I_p_* inhibition by Pm when applied as indicated in panels (**A_1_**–**A_3_**,**B_1_**–**B_3_**), at −60 mV (on the left) and +40 mV (on the right). (*) indicates significant differences between *I_p_* inhibition elicited by 1 and 5 µM Pm (*p* < 0.05, *t*-test). (#) denotes significant differences, for each Pm concentration, among the percentages of *I_p_* inhibition elicited by ACh and Pm co-application and other Pm-application protocols, at the same holding potential (*p* < 0.05, ANOVA and Bonferroni *t*-test). Each column is the average of 5–11 and 5–17 oocytes, for −60 mV and +40 mV, respectively.

As aforementioned, when ACh is co-applied with Pm, up to 1 µM, *I_ACh_* is mostly inhibited by open-channel blockade. This blockade is voltage-dependent and requires the channel opening by the agonist. However, increasing Pm to 5 µM, unraveled an additional blocking mechanism, which could not be precluded by applying positive-voltage pulses (Figure 5B,C). To get a deeper understanding of Pm effects on nAChRs, a set of experiments were performed, at both negative and positive potentials, combining different timing of Pm application. Oocytes were clamped at either −60 mV or +40mV and *I_ACh_*s were elicited by 32 s superfusion of 10 µM ACh alone (Figure 8, black recordings) or together with either 1 or 5 µM Pm (Figure 8, orange and purple recordings, respectively), following different Pm-application protocols: (i) Pm co-applied with ACh (Figure 8A_1_,B_1_), (ii) Pm pre-applied for 12 s before bathing the cell with ACh alone (Figure 8A_2_,B_2_), and (iii) 12 s pre-application of Pm followed by its co-application with ACh (Figure 8A_3_,B_3_).

At 1 µM Pm, direct co-application of Pm and ACh decreased *I_ACh_* roughly 40% when the membrane potential was held at −60 mV, but it had almost no effect at +40 mV (Figure 8A1,C). When Pm was pre-applied before superfusing ACh to the cell, there was a slight *I_ACh_* inhibition (roughly 10%), but only at the negative potential (Figure 8A_2_), indicating that some Pm molecules bound at the channel pore pathway. Accordingly, Pm pre-application followed by its co-application with ACh blocked *I_ACh_* roughly the same degree as merely its co-application with the agonist (compared to Figure 8 panels A_1_ and A_3_). When Pm concentration increased to 5 µM, its co-application with ACh, at −60 mV, significantly enhanced *I_ACh_* inhibition, as compared to that elicited by 1 µM Pm (Figure 8 B_1_,C) and, interestingly, it also blocked nAChRs at +40 mV (Figure 8B_1_,C). Just pre-application of 5 µM Pm inhibited *I_ACh_* both at positive and negative potentials, although the *I_ACh_* blockade at +40 mV was roughly one third of that elicited at −60 mV (20.4 ± 1.7%, *n* = 8, versus 7.0 ± 2.2%, *n* = 6, for −60 and +40 mV, respectively; *p* < 0.01, *t*-test; Figure 8C). Thus, Pm could also block resting nAChRs and their recovery, after Pm washed out, was very slow, requiring over 30 s for a full recuperation (see Figure 8A_2_,B_2_). When 5 µM Pm was pre-applied and then co-applied with ACh, the *I_ACh_* inhibition, at either −60 or +40 mV, was quite similar to that evoked by solely Pm and ACh co-application at the respective potential, (Figure 8B_1_,B_3_,C), likewise as when applying 1 µM Pm (see above).

### 2.7. Molecular Docking and Dynamics Simulations of Pm-nAChR Complexes

**Figure 9:**
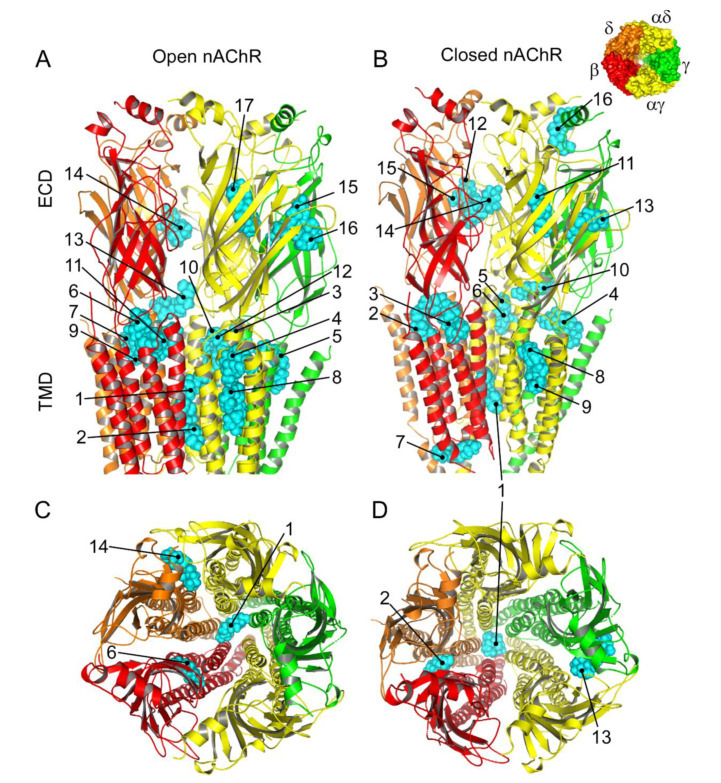
Predicted binding sites for Pm-nAChR complexes. (**A**,**B**) Lateral view of the nAChR displaying the main Pm (labelled in cyan) clusters bound to the open (**A**) and closed (**B**) conformations. The predicted loci are numbered consecutively, beginning in the transmembrane (TMD) and later in the extracellular (ECD) domain. (C, D) Top view of the nAChR (from the synaptic cleft) displaying representative Pm clusters binding to residues located within the channel pore, TMD, and ECD in the open (**C**) and closed (**D**) conformations. The inset, in the upper right corner, displays the nAChR subunits with their color code.

Several docking solutions were found when performing 999 Pm runs for each nAChR conformation. The choice of the best solutions was based on the combination of two criteria: the best calculated binding energy (ΔG ≤ −9.3 kcal/mol) and the most crowded clusters. Most Pm clusters were located at the TMD, in both the open (1–13) and the closed (1–10) conformations (Figure 9A,B and Supplementary Table S1). TMD clusters were located at either intra- or inter-subunit crevices, without a clear preference for any subunit (see Supplementary Table S1). Noticeably, three solutions situated Pm interactions within the channel pore; two of them were in the open conformation (Pm 1 and Pm 2; Figure 9A,C), whereas the other was found in the closed-state (Pm 1, Figure 9B,D and Supplementary Table S1). Although Pm is a highly hydrophobic molecule (Figure 1A), and therefore it should preferentially distribute within the lipid bilayer, several clusters (sites 14–17 and 11–16 in the open and closed structures, respectively) localized at ECD. Some of these clusters were sited at the interface of the α-γ and α-δ subunits in both states, close to the orthosteric binding sites (Figure 9A,B and Supplementary Table S1).

**Figure 10:**
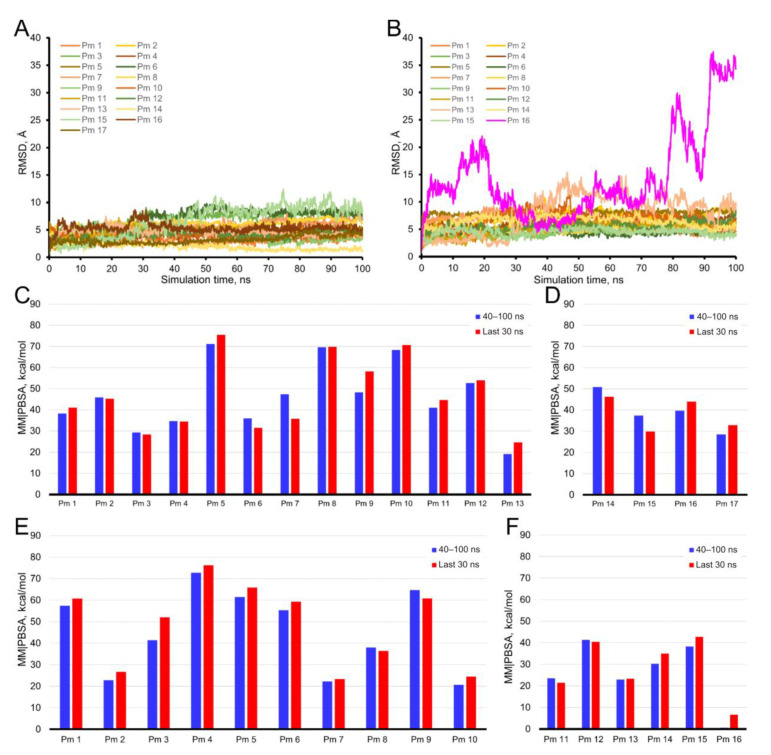
Analysis of MD simulations for Pm bound to the ECD and TMD of nAChR in open and closed conformations. Panels (**A**) (open conformation) and (**B**) (closed conformation) display the trajectory through a 100 ns simulation of a Pm molecule sited in each main cluster at the TMD and the ECD, as numbered in Figure 9A,B. Panels (**C**) (TMD, open), (**D**) (ECD, open), (**E**) (TMD, closed), and (**F**) (ECD, closed) display the free energy analysis (MM|PBSA) of the Pm-nAChR complexes at two MD trajectory intervals (40 to 100 ns and the last 30 ns). YASARA-calculated binding energy provides positive values when the predicted binding is strong and stable, whereas negative values indicate unstable or no binding.

Molecular dynamics simulations for each Pm cluster bound to the TMD and ECD of lipid-reconstituted nAChR is presented in Figure 10, for the open (panel A) and closed (panel B) conformations. Each plot displays the trajectory of a Pm molecule from the selected clusters through a 100 ns simulation. Root mean square deviation (RMSD) values lower than 10 Å correspond to rearrangements of the Pm into their binding site through the simulation period, since Pm molecule is circa 12.5 Å long. Remarkably, all Pm molecules, except Pm 16 of the closed-state (Figure 10B), remained steadily bound at their initial binding site on the nAChR through the 100 ns simulation. 

To further verify the stability of the Pm-nAChR complexes, we carried out an evaluation of the MM/PBSA, which estimates the free energy of the binding of small ligands to biological macromolecules. MM|PBSA average binding energies were computed for Pm bound to both the open (Figure 10C,D, for TMD and ECD, respectively) and closed (Figure 10E,F for TMD and ECD, respectively) conformations. Several aspects should be highlighted regarding these results: (i) for all Pm molecules, the average values calculated for the last 60 ns are very close to those obtained for the last 30 ns, which suggests that the system stabilizes during the first 40 ns of simulation, (ii) the binding free energy values for the different Pm molecules are quite similar, regardless of whether we consider the open- or closed-states (Figure 10C–F), (iii) Pm binding to ECD, both for the closed- and open-states, is commonly weaker than its binding to TMD, and (iv) high MM|PBSA values (as much as 70 kcal/mol) were reached for some Pm binding sites (see Figure 10C,E).

**Figure 11:**
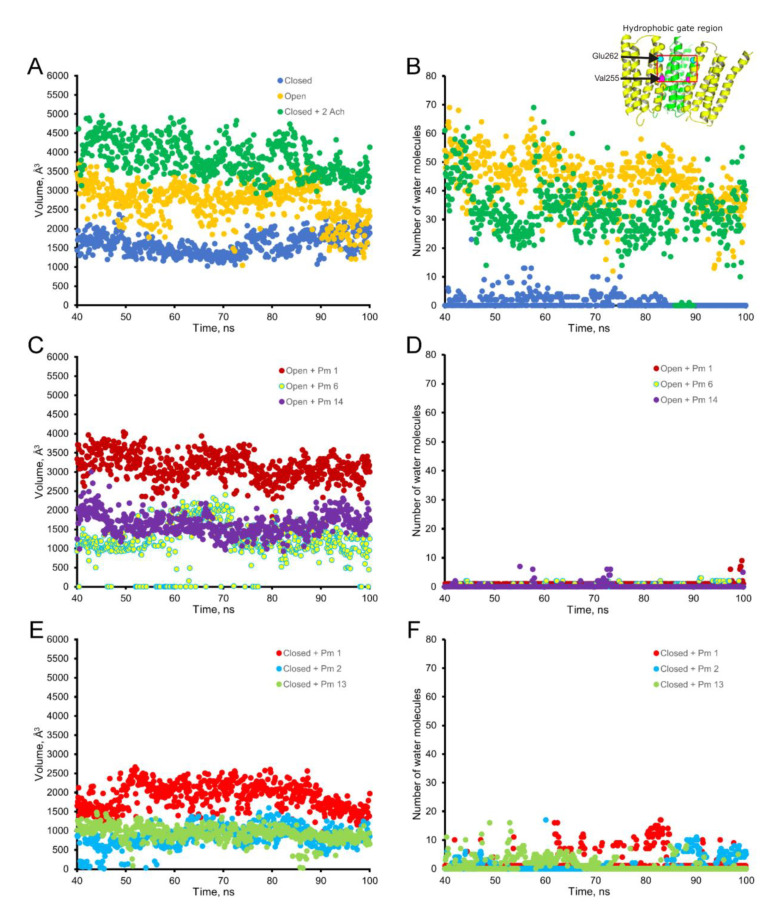
Empty volume (left panels) and number of water molecules (right panels) within the hydrophobic gate of the nAChR pore region (between Val255 and Glu262 of the alpha subunit; see inset in the upper right corner), through the period of 40 to 100 ns of MD simulations. Top panels show the empty volume (**A**) and the number of water molecules (**B**) at the hydrophobic gate region in control nAChR (in the absence of Pm), both in the open (yellow) and the closed (blue) conformations; also, it displays the effect of ACh on the closed conformation (green). Middle panels (**C**,**D**) display the effect of Pm on these parameters when located at representative sites of the nAChR in the open conformation: within the channel pore (Pm 1), at the TMD (Pm 6), and the ECD (Pm 14). Lower panels (**E**,**F**) demonstrate the effect of Pm at representative loci of the nAChR in the closed conformation: inside the channel (Pm 1), at TMD (Pm 2), and at ECD (Pm 13).

Channel pore hydration analysis displays a continuous water column when the nAChR is in the open-state, whereas it is interrupted in the upper half of the M2 transmembrane segment in the closed-state, indicating the presence of a hydrophobic gate [29]. This partial dehydration of the extracellular half of the channel pore interrupts the connection between the extra- and intracellular media and is a consequence of the conformational changes of the M2 helices [29]. Consequently, the number of water molecules into the extracellular half of the pore region might be considered a gating indicator. The effect of Pm binding to different sites of the nAChR on both the empty volume of the channel-pore and the number of water molecules within this region are displayed in Figure 11, for both the open and the closed-states. In the absence of Pm (i.e.**,** control conditions (Figure 11A,B)), the average of water molecules located within the hydrophobic gate was 48 for the open-state whereas for the closed-state was only 4. Even more, in the closed-state, most of the time there were no water molecules within this channel-pore region. The empty volume of the hydrophobic gate region was roughly 2900 Å^3^ and 1500 Å^3^ for the open- and the closed-state, respectively. MD simulations also included the effect of ACh docked to the nAChR at the two orthosteric sites in the closed-state, in absence of Pm. As expected, ACh switched the channel to an open-like conformation, as evidenced by an increase in the number of water molecules as well as a marked increase in the empty volume of the pore at this region, up to values similar to those found for the open channel (Figure 11A). Additional MD simulations were addressed to show the changes elicited by Pm binding to open and closed nAChRs in both empty volume and number of water molecules within the hydrophobic-gate region. Pm effects on the open nAChR are illustrated in Figure 11C,D, which displays the structural changes elicited when Pm binds to three representative sites: Pm 1 (located shallow within the channel pore), Pm 6 (sited at TMD, in a crevice of the β subunit), and Pm 14 (bound to the ECD, at the interface between α-δ subunits; see Figure 9C). Noticeably, all of these binding sites decreased the number of water molecules to almost none (Figure 11D), and both Pm 6 and Pm 14 markedly decreased the empty volume of the pore (Figure 11C). By contrast, Pm 1, located within the hydrophobic gate, did not reduce the empty volume of this zone (≈3100 Å^3^; Figure 11C), most likely because Pm bound to this channel-pore region precluded its narrowing.

Regarding the closed-state, Pm 1 (located inside the pore), Pm 2 (docked to the TMD at crevices in the interface between β-δ subunits) and Pm 13 (sited at the ECD, at the interface between α-γ subunits) are representative examples of the structural effects mediated by Pm when binding to different nAChR regions (see Figure 9D). As compared to the control situation, Pm 1 neither affects the number of water molecules at the hydrophobic gate region nor the empty volume of this zone, which remained close to 1800 Å^3^ (Figure 11E,F). Interestingly, the effects of Pm 2 and Pm 13 on closed nAChRs were quite similar (Figure 11E,F), even though their binding sites were located far away from each other. In fact, both Pm 2 and Pm 13 decreased the empty volume of the pore and prevented the presence of water molecules at the hydrophobic gate. 

## 3. Discussion

A broad number of medicinal plants have been used for centuries as therapeutic tools in TCM. However, neither the active compounds of many of these plants nor their mechanisms of action are yet well-understood. We have now studied the effect of Pm, an isosteroidal alkaloid considered one of the main bioactive molecules of *F*b, on nAChRs.

Remarkably, Pm decreased *I_ACh_* in a dose-dependent manner, with an IC_50_ in the low micromolar range (circa 3 and 1 µM for *I_p_* and *I_ss_*, respectively; Figure 1). These IC_50_s are markedly lower than the values previously reported for Pm blockade of voltage-dependent potassium channels. Thus, Pm IC_50_ for blocking Kv1.2 was 472 µM, it was 354 µM for Kv1.3 (142 µM if measured 150 ms after the current peak), and even much higher for Kv1.4 to Kv1.8 channels [10]; additionally, Pm inhibited the potassium channel hERG, with an IC_50_ of 44 µM, most likely by enhancing its inactivation [11]. Pm also blocked the Nav1.7 channel (IC_50_, 47 µM), demonstrating use-dependent inhibition, like the blocking mechanism of lidocaine on this channel [10]. Pm effects on hERG channels are particularly remarkable, since these channels play a key role in myocardial repolarization and, therefore, their inhibition might cause serious cardiac arrhythmias. Despite this, humans have used *F*b as a therapeutic herb for centuries, being considered safe for consumption. Consequently, intake of *F*b should not markedly affect the activity of hERG channels, despite that Pm, one of its main bioactive compounds, blocks these channels with an IC_50_ of 44 µM [11]. Nevertheless, Pm has a very low oral bioavailability [9] and thus its plasma concentration after *F*b intake should be fairly low. Actually, Pm content in bulbs of *Fritillaria ussuriensis* and *thunbergii* ranged from 0.58 to 1.2 mg/g and oral administration of powder from these *Fritillaria* plants to dogs (1 g/kg) raised Pm plasma concentration to a maximum of 100–200 nM [30]. Accordingly, a similar Pm bioavailability was reported after oral administration of *Fritillaria thunbergii* extracts in rats, with peak plasma concentrations of Pm of roughly 100 nM [31]. These Pm concentrations are several orders of magnitude lower than the IC_50_s reported for sodium or potassium channels (including hERG), muscarinic receptors, or acethylcholinesterase [8]. Interestingly, submicromolar Pm concentrations elicit a significant inhibition of nAChRs (roughly 20%; Figure 1) and therefore this family of LGIC might be relevant targets of its actions. Of note, we have assessed Pm actions on muscle-type nAChRs, because they are the prototype member of this family of receptors, but Pm might have different affinities for related receptors, as the homomeric α7. In fact, α7 nAChRs are largely expressed in non-neuronal tissues, including macrophages, and exert powerful anti-inflammatory actions [16]. Moreover, other nAChR subtypes, such as α4β2 and/or α9α10, may also play a role in modulating inflammatory processes and even in chronic pain [15]. Noticeably, Pm displayed a differential affinity for different LGICs, even of the same family. Thus, whereas Pm inhibits muscle-type nAChRs with and IC_50_ close to 1 µM, GABA_A_ receptors were almost not affected by Pm at concentrations up to 100 µM.

Pm exerted a non-competitive inhibition on muscle-type nAChRs, since *I_ACh_*s halved when co-applying Pm, at its IC_50_, with different ACh concentrations (Figure 2A,B). Several blockade mechanisms seem involved in this non-competitive inhibition of nAChRs by Pm, which is coherent with the multiple binding sites predicted by the docking simulations. First, open-channel blockade, as *I_ACh_* inhibition by Pm was voltage-dependent, the more hyperpolarized the cell, the more pronounced the blockade (Figure 5). Actually, this is what would be expected for a positively charged molecule plugging the channel pore. In agreement with this, the docking assays predicted some Pm clusters located within the channel pore, both in the open and closed conformations (Figure 9). Thus, the open-channel blockade of nAChRs mediated by Pm resembles that mediated by some LAs, such as lidocaine [24] or tetracaine [26]. Nevertheless, the kinetics of open-channel blockade of nAChR elicited by Pm was rather slow. Thus, at −60 mV, the τ of open channel blockade elicited by 1 µM Pm (close to its IC_50_) was over 2 s and even by 5 µM Pm (eliciting roughly 70% of *I_ss_* blockade) the time constant was above 1 s (Figure 7). In contrast, at the same membrane potential, 0.7 µM tetracaine (close to its IC_50_) blocked open nAChRs with a time course of roughly 300 ms [26]. These differences in time constants between Pm and tetracaine are most likely related to their distinct molecular sizes (Pm molecular weight is over 60% greater than that of tetracaine). Second, Pm enhanced nAChR desensitization, as evidenced by: (i) acceleration of *I_ACh_* decay when co-applying ACh with Pm at concentrations of 0.5 µM, or above (Figure 3A_2_,B_2_), and (ii) shortening of the *I_ACh_* aTtp, which correlated with the acceleration of *I_ACh_* decay (Figure 3A_1_,B_1_). Likewise, lidocaine decreased the *I_ACh_* aTtp only at concentrations that enhanced *I_ACh_* decay [24]. Furthermore, DMA, a lidocaine analog, both sped up *I_ACh_* decay and shortened the aTtP [25]. By contrast, diethylamine (DEA), a lidocaine analog that mainly blocks nAChR by open channel blockade neither accelerates *I_ACh_* decay nor decreases aTtP [21], and (iii) deceleration of *I_ACh_* deactivation, which was dependent on Pm concentration and displayed a good correlation with the rate of *I_ACh_* decay (Figure 4A, B). The deceleration of *I_ACh_* deactivation when Pm remained in the solution strongly supports that Pm enhanced nAChR desensitization, since desensitized nAChRs display higher affinity for the agonist [22,26,27]. Third, Pm elicited the blockade of resting (closed) nAChRs. This effect was unraveled by applying Pm before challenging the cells with ACh alone. This protocol, which allowed Pm to act only on resting (closed) nAChRs, elicited a mild nAChR blockade, mostly at negative potentials. Actually, at positive potentials, *I_ACh_* only decreased by Pm pre-application when rising its concentration to 5 µM (Figure 8). In agreement with this, *I_ACh_* inhibition by 5 µM Pm was slightly higher when Pm was pre-applied and then co-applied with ACh than when solely co-applied with ACh (Figure 8C). 

Our virtual docking and MD simulations used as a template the structure of *Torpedo* nAChRs in the open and closed conformations released by Unwin’s group [32,33]. However, a new structural model of the nAChR from *Torpedo*, at higher resolution and stabilized in the closed conformation by α-bungarotoxin, has been recently disclosed [34]. The new structural model (pdb entry 6UWZ) share large similarities with the Unwin’s model for the closed conformation, though there are certain differences between them. Mostly, they differ in the upper portion of the pore, which is more constricted in the new model, and in the δ subunit arrangement [35]. It seems that these discrepancies arise because of differences in the lipid matrix surrounding the nAChR. Actually, cholesterol interactions with the nAChR are apparently essential for stabilizing its structure and the absence of cholesterol (as in the model of Rahman et al. [34]) leads to a more compact arrangement of TM helices (displacement of helices circa 1–3 Å; [35]). Noticeably, the major lipid present in electroplax membranes rich in nAChRs is cholesterol [36] and purified nAChRs from *T. marmorata* and *E. electricus* interact preferentially with cholesterol rather than with either phospholipid monolayers or other sterols [37]. Moreover, nAChRs in native electroplax membranes are arranged as dimers, linked by their δ-subunits. This interaction between neighboring nAChRs might account for the differences in the δ-subunit between both structural models since dimers were reduced to monomeric receptors in the Rahman’s model. In fact, we chose for our structural studies Unwin’s models because of: (i) the structures for the open and closed conformations are available, (ii) the nAChR is present in their original membrane, and (iii) we have significant experience correlating structural and functional results using these commonly accepted models; actually, Unwin’s models have so far provided a coherent correlation with our functional results [21,25,26,28].

The virtual docking assays predicted Pm binding to the nAChR at different sites of the TMD and ECD in the open conformation. Most Pm clusters were located at the TMD, at inter- and intra-subunit crevices, although some of them located into the channel pore (Figure 9A,C). The binding energies estimated for these clusters were rather high (from −9.3 to −12.87 kcal/mol; see Supplementary Table S1), pointing out that Pm has a high affinity for the nAChR. Remarkably, MD simulations of nAChRs in the open conformation indicate that Pm binding to the nAChR either at the TMD (i.e., cluster 6) or at the ECD (i.e., cluster 14) markedly decreased both the volume and the number of water molecules at the hydrophobic gate region of the channel pore (Figure 11). Furthermore, virtual docking and MD assays pointed out that Pm also interacts with the nAChR in the resting conformation, binding to residues located at both the TMD and the ECD (Figure 9B,D and Figure 11E,F). Interestingly, the structural changes of the nAChR induced by Pm, as predicted by docking and MD assays, are in good agreement with the functional changes elicited by Pm on this receptor. Actually, the structural and functional results can be correlated as follows: (i) the high binding energies computed accounted for the high potency of Pm blocking nAChRs, (ii) Pm interaction with residues located within the channel pore should trigger the open-channel blockade, (iii) Pm binding to different sites at the nAChR might explain both its heterogeneity of actions on nAChRs and the effect-dependence on Pm concentration, and (iv) Pm binding to the nAChR in the closed conformation might underlie the blockade of resting nAChR. Consequently, the good correlation between structural simulations and electrophysiological results strongly suggests that Pm actually blocks nAChRs by the different aforementioned mechanisms.

Since *F*b has been used as therapeutic herb for thousands of years, there is a strong support for its beneficial effects and its weak (or lack) of toxicity. However, neither the identity of all its bioactive compounds nor their mechanisms of action are yet well known. Now, we report here that Pm, considered one of the main bioactive compounds from *Fritillaria*, exerts a powerful inhibition of muscle-type nAChRs, which, as far as we know, is the first report demonstrating that Pm might modulate LGICs, besides acting on other targets as voltage-dependent channels or metabotropic receptors. It remains to be unraveled if Pm might modulate other nAChRs, including the homomeric α7, which is broadly expressed in immune cells and has been related to powerful anti-inflammatory actions [16]. Furthermore, both mecamylamine, a non-competitive antagonist of α7 nAChRs, and 1-ethyl-4-(3-(bromo)phenyl)piperazine, which promotes α7 desensitization, reduce pro-inflammatory responses [38]. We have now demonstrated that Pm inhibits muscle-type nAChRs and it can be hypothesized that it might modulate, though in a different way or with a different potency, either α7 or other nAChRs. In this regard, lidocaine exerted similar inhibitory actions on muscle-type nAChRs [24] and on neuronal nAChRs expressed in autonomic-ganglia neurons [39]. Alternatively, it could be that different bioactive compounds from *F*b accounted for its anti-inflammatory actions, as anthocyanin pigments, which are flavonoids present in *F*b [40]. Noticeably, some flavonoids act as positive allosteric modulators of α7 nAChRs, although without affecting desensitization [6], and their enhancement of α7 activity has been proposed as a therapeutic strategy for inflammatory disorders [41].

## 4. Materials and Methods

### 4.1. Oocyte Microinjection with Either Proteoliposomes Bearing nAChRs or Rat-Brain Synaptosomal-Enriched Membranes Bearing GABA_A_Rs

Adult female *Xenopus laevis* (purchased from Centre National de la Recherche Scientifique, Montpellier, France and European Xenopus Resource Centre (EXRC), Portsmouth, UK) were immersed in cold 0.17% tricaine methanesulfonate (MS-222) for 20 min and a piece of ovary was drawn out aseptically. Animal handling was carried out in accordance with the guidelines for the care and use of experimental animals adopted by the European Union (European Communities Council Directive of 22 September 2010, 2010/63/UE), and the animal protocol was approved by the Ethic Committee of Universidad de Alicante. Stage V and VI oocytes were isolated and their surrounding layers removed manually. Cells were kept at 15–16 °C in a modified Barth’s solution (88 mM NaCl, 1 mM KCl, 2.40 mM NaHCO_3_, 0.33 mM Ca(NO_3_)_2_, 0.41 mM CaCl_2_, 0.82 mM MgSO_4_, 10 mM HEPES (pH 7.4), 100 U/mL penicillin and 0.1 mg/mL streptomycin) until used.

*Torpedo marmorata* nAChRs were purified and reconstituted in asolectin lipids, at a final protein concentration of 0.3–1.2 mg/mL, as previously reported [42]. Oocytes were microinjected with 100 nL of an aliquot of reconstituted nAChRs. In some experiments, gamma-aminobutyric acid GABA_A_Rs were microtransplanted to the *Xenopus* oocyte membrane from rat-brain synaptosomal-enriched membranes, as previously described [21].

### 4.2. Two-Electrode Voltage-Clamp Recordings in Oocytes

Membrane currents were recorded 16–72 h after proteoliposome injection, as previously reported [21,23]. Briefly, oocytes were continuously superfused with normal frog Ringer’s solution (115 mM NaCl, 2 mM KCl, 1.8 mM CaCl_2_, 5 mM HEPES, pH 7.0) supplemented with 0.5 μM atropine sulfate (normal Ringer with atropine, ANR) to block any muscarinic response [43]. The membrane potential was held at −60 mV, unless otherwise stated. Membrane currents elicited by ACh, either alone or co-applied with Pm, were low-pass filtered at 30–1000 Hz and, after sampling at fivefold the filter frequency (Digidata series 1440A and 1550; Axon Instruments, Foster City, CA, USA), recorded on two PC-computers, using the WCP v. 4.8.6 package developed by J. Dempster (Strathclyde Electrophysiology Software, University of Strathclyde, Glasgow, UK) and AxoScope v. 10.0.0.60 (Molecular Devices Corporation, Sunnyvale, CA, USA).

### 4.3. Experimental Design

Experimental procedures were similar to those previously used to study the effects of either acetylcholinesterase inhibitors [44,45] or LAs on nAChRs [24,26,28]. Briefly, Pm concentration-*I_ACh_* inhibition relationship was determined by measuring *I_ACh_*s evoked by 10 μM ACh alone or together with different Pm concentrations. For competition assays, ACh concentration- versus *I_ACh_* amplitude curves were obtained by bathing nAChR-bearing oocytes with increasing ACh concentrations either alone or together with Pm at a concentration close to its IC_50_. *I_ACh_*s were normalized to the maximum *I_ACh_* evoked by ACh alone, and a sigmoid curve was fitted to these values (see equation 2 below). To allow nAChRs to recover from desensitization, the interval between consecutive ACh applications was, at least, 5 min. Blockade of resting nAChRs by Pm was assessed by the pre-application of Pm (1–5 µM) for 12 s before challenging the cell with ACh alone. The voltage dependence of the *I_ACh_* inhibition by Pm was determined by giving series of 800–1200 ms voltage pulses (from −120 to +60 mV, in 20 mV steps) to the oocyte before ligand superfusion and during the *I_ACh_* plateau elicited by 10 μM ACh, either alone or co-applied with either 1 or 5 µM Pm. The time course of nAChR blockade by Pm and its recovery were assessed by applying Pm (1–5 µM) for 20 s during the *I_ACh_* plateau elicited by a 50 s pulse of 10 µM ACh. Furthermore, to better resolve the time course of open-channel blockade of nAChR by Pm, a 2 s voltage pulse, from −60 to +40 mV, was applied during the *I_ACh_* plateau elicited by 10 µM ACh either alone or co-applied with 1 or 5 µM Pm and the kinetics of *I_ACh_* blockade by Pm after jumping back from +40 to −60 mV was computed.

Oocytes previously injected with synaptosomal membranes bearing GABA_A_R were superfused with 1 mM GABA alone or together with Pm (10–100 μM) to assess the Pm effects on GABA elicited currents (*I_GABA_*).

### 4.4. Data Analysis and Statistical Procedures

Inhibition curves were determined by measuring the *I_ACh_* evoked by 10 μM ACh in the presence of different concentrations of Pm. The *I_ACh_*s (both at the peak, *I_p_*, and 20 s later, *I_ss_*) elicited in the presence of Pm were normalized to the *I_ACh_* evoked by ACh alone. A logistic curve was fitted to the data with the OriginPro 8 software (OriginLab Corp., Northampton, MA, USA), using the following Equation (1):

(1) $$I_{ACh+Pm}=\left(\;\frac{I_{ACh}max-I_{ACh}min}{1+{\left(\left[Pm\right]/IC_{50}\right)}^{n_{H}}}\right)+I_{ACh}min$$

where *I_ACh_*_+ *Pm*_ is the *I_ACh_* amplitude elicited by co-application of 10 μM ACh with Pm at a given concentration (*[Pm]*), *I_ACh_max* and *I_ACh_min* are the maximum and minimum *I_ACh_*s recorded, respectively, *IC_50_* is the Pm concentration required to halve the *I_ACh_max*, and *n_H_* is the Hill coefficient.

The rate of desensitization (*I_ACh_* decay) was determined by fitting to a single exponential function the *I_ACh_* elicited by 10 or 100 µM ACh, either alone or co-applied with different Pm concentrations [26]. The apparent time-to-peak (aTtP) was determined as the time elapsed from *I_ACh_* onset to the *I_p_* elicited by ACh either alone or with Pm. We have called this parameter as “apparent” to indicate that these values do not necessarily reflect “real” time-to-peak values of nAChR activation, but those observed in our experimental conditions.

To characterize the pharmacological profile of nAChR blockade by Pm, nAChRs were activated by different concentrations of ACh alone, or co-applied with Pm at roughly its IC_50_. The following form of the Hill Equation (2) was used to fit dose-response data:

(2) $$I/I_{ACh}max={\left[1+{\left(EC_{50}/\left[ACh\right]\right)}^{n_{H}}\right]}^{-1}$$

where *I* is the *I_ACh_* amplitude elicited at a given concentration of ACh (*[ACh]*) applied either alone, or together with Pm, *EC_50_* is the agonist concentration required to halve the maximum *I_ACh_*, and *I_ACh_max* and *n_H_* are as in Equation (1).

Net *i/v* curves for *I_ACh_* were computed by subtracting, for each voltage, the steady-state currents attained in ANR (measured at the last 100 ms of the pulse) from the corresponding currents recorded in the presence of 10 µM ACh either alone or together with Pm. These net *I_ACh_* values were normalized, for each oocyte, to the *I_ACh_* at −60 mV elicited by ACh alone.

To determine the rate of open-channel blockade by Pm, the oocyte was superfused with either 1 or 5 µM Pm at the plateau of the *I_ACh_* elicited by 10 µM ACh. A single exponential function was fitted to the *I_ACh_* decrease elicited in the presence of Pm. The time constant (τ) of the *I_ACh_* decay was computed by using the OriginPro 8 software. The same procedure was followed to determine the kinetics of *I_ACh_* recovery upon Pm withdrawal. A similar fitting method was used to estimate the kinetics of *I_ACh_* deactivation (i.e., the time course followed by the *I_ACh_* to return to the baseline level after removal of 100 µM ACh either alone or together with 1 or 5 µM Pm).

Unless otherwise specified, values presented are the mean ± standard error of the mean (SEM), “*n*” indicates the number of oocytes, and “*N*” is the number of oocyte-donor frogs from which the data were obtained. When comparing two-group means of normally distributed values, the Student’s *t*-test was used; otherwise, the Mann-Whitney rank-sum test was applied. Among-group differences were determined by the analysis of variance (ANOVA), and mean differences for each pair of groups were determined with the Bonferroni *t*-test. The one-sample *t*-test was used to compare the mean of an experimental group with a specified value. A significance level of *p* < 0.05 was considered in all cases.

### 4.5. Molecular Docking and Dynamics Simulations

#### 4.5.1. Pm-nAChR Docking Simulations

The structures of the nAChR (*Torpedo marmorata*), both for the tense (closed, 2BG9 [32]) and for the more relaxed (open, 4AQ9 [33]) conformations, were obtained from the Research Collaboratory for Structural Bioinformatics (RCSB). Molecular docking simulations of Pm on the surface of nAChR have been carried as described elsewhere [21,25,46]. A total of 999 flexible docking runs were set and clustered (i.e., two docked compounds were considered to belong to different clusters when the ligand root-mean-square deviation of their atomic positions was greater than 7 Å around certain hot spot conformations). The YASARA software calculated the Gibbs free energy variation (∆G, kcal/mol) with more negative values, indicating stronger binding. The Pm molecule for each cluster with a more negative ∆G value (stronger binding) was used in the molecular dynamics simulations.

#### 4.5.2. Molecular Dynamics Simulations (MD) of Lipid Reconstituted nAChR-Pm Complex

Pm molecules with the best binding to the nAChR of each cluster, were used as the starting point for independent MD simulations. Before starting each simulation, the Pm-AChR complex was reconstituted into a lipid bilayer (phosphatidyl-choline:phosphatidyl-serine, 80:20 for both bottom and top membrane side) using the YASARA macro md_runmembrane.mcr. The simulation cell was allowed to include 20 Å surrounding the protein, which were filled with water at a density of 0.997 g/mL. Initial energy minimization was carried out under relaxed constraints using the steepest descent minimization. Simulations were performed in water at constant conditions of pressure (1 bar) and temperature (25 °C). To mimic physiological conditions, counter ions were added to neutralize the system, Na^+^ or Cl^−^ were added as replacement of water to give a total NaCl concentration of 0.9%, and pH was maintained at 7.4. The pK_a_ was computed for each residue according to the Ewald method [47]. All simulation steps were run by a preinstalled macro (md_run.mcr) within the YASARA suite. Data were collected every 100 ps for 100 ns. Each simulation was carried out in the high-performance computing Linux cluster of the Centro de Computación Científica (CCC-UAM). The measurement of the number of water molecules in the upper half of the channel pore was carried out using the macro md_analyze.mcr from YASARA. The Molecular Mechanics/Poisson-Boltzmann surface area (MM/PBSA) was implemented with the YASARA macro md_analyzebindenergy.mcr, to calculate the binding free energy with the solvation of the ligand, complex, and free protein, as previously described [48,49].

Measurements of cavity volume inside the nAChR were performed with 3V Voss Volume Voxelator [50] installed locally, and UCSF Chimera v1.15, build 42,209 [51]. The volumes were set with 3V by transforming the protein pdb coordinates to xyz format with the “pdb_to_xyzr” program. Then, the identification of the main, largest cavity was performed with the “AllChannel.exe” program, using an external probe radius of 10 Å, an internal probe radius of 3.3 Å, and restricting the number of channels to 1. This provided an mrc map containing a three-dimensional grid of voxels, each with a value corresponding to cavity volume. Molecular graphics Chimera software, developed by the Resource for Biocomputing, Visualization, and Informatics at the University of California, San Francisco, was used to measure volumes using the commands “vop zone” and “measure volumes”. The sub-volumes were measured by selecting the residues of each subunit that outline the upper part of the pore (α1: 255–261, β: 261–267, δ: 269–275, α2: 255–261, and γ: 263–269; see inset of Figure 11). Values were provided in Å^3^.

### 4.6. Drugs

ACh, atropine sulphate, Pm, GABA, MS-222, DMSO, penicillin, and streptomycin were from Sigma-Aldrich-Merck (Darmstadt, Germany). HEPES was obtained from Acros Organics (Geel, Belgium). Other reagents of general use were purchased from Scharlau Chemie SA (Barcelona, Spain). Pm solutions were prepared from a 10 mM stock solution in DMSO. All solutions were made in ANR just before each application.

## 5. Conclusions

A plethora of therapeutic herbs are used in TCM to treat different illnesses. *Fritillaria* plants are included among them, as they are often used to treat asthma, to alleviate cough, and as anti-inflammatory and analgesic therapy. We have now assessed the effect of Pm, an isosteroidal alkaloid found in *F*b, on purified and reconstituted nAChRs microtransplanted to the *Xenopus* oocyte membrane. Pm exerted a powerful inhibition of muscle-type nAChRs acting by different mechanisms: (i) open-channel blockade, as evidenced by its voltage-dependent inhibition of *I_ACh_*, (ii) enhancement of nAChR desensitization, as supported by three observables: first, the acceleration of the *I_ACh_* decay, second, the slowing down of *I_ACh_* deactivation in the presence of Pm, and third, the shortening of the *I_ACh_* aTtP; and (iii) closed (resting) channel blockade, which was demonstrated by the *I_ACh_* inhibition elicited by Pm when it was pre-applied before challenging the cell with ACh alone (thus Pm action was restricted to resting nAChRs). Furthermore, virtual docking and MD assays of Pm interactions on nAChRs, both in the open and closed conformations, predicted that Pm interacts with high affinity at different sites on this receptor, which seems consistent with the variety of functional effects observed. Moreover, the observed binding sites support fairly well the functional effects of Pm on nAChRs. 

As far as we know, this is the first report demonstrating that Pm modulates LGICs, and moreover, its effects seem quite selective on nAChRs, since other receptors of the same family, such as GABA_A_R, were unaffected by Pm. Considering that Pm plasma levels after oral administration of *F*b are rather low (in the submicromolar range) and that nAChRs constitute a high affinity target for Pm, it turns out that its anti-inflammatory and analgesic effects could be mediated through this interaction, given that nAChRs mediate potent anti-inflammatory effects.

## Supplementary Materials

The following materials are available online at https://www.mdpi.com/article/10.3390/ijms222011287/s1.

## Abbreviations

ACh	acetylcholine
ANR	normal Ringer solution with atropine
aTtP	apparent time to *I_ACh_* peak
CI	confidence interval
DEA	diethylamine
DMA	2,6-dimethylaniline
EC	extracellular
*F*b	*Fritillaria* bulbs
GABA_A_R	gamma-aminobutyric acid receptor type A;
hERG	human ether-a-go-go related gene
*I_ACh_*	ACh-elicited current
IC	intracellular
*I_GABA_*	GABA-elicited current
*I*_p_	*I_ACh_* amplitude at the peak
*I_ss_*	*I_ACh_* amplitude 20 s after *I*_p_
LA	local anesthetic
LGIC	ligand-gated ion channel
MD	molecular dynamics simulation
MM|PBSA	molecular mechanics/Poisson-Boltzmann surface area
MS-222	ethyl 3-aminobenzoate methanesulfonate
*n*	number of oocytes
*N*	number of oocyte-donor frogs
nAChR	nicotinic acetylcholine receptor
Pm	peimine
RMSD	root-mean-square deviation
SEM	standard error of the mean
TCM	Traditional Chinese medicine
TM	transmembrane spanning-segment

## References

[1] Wang D., Zhu J., Wang S., Wang X., Ou Y., Wei D., Li X. (2011). Antitussive, expectorant and anti-inflammatory alkaloids from Bulbus *Fritillariae Cirrhosae*. Fitoterapia.

[2] Du Q., Wang D., Wang S. (2016). The pharmaceutical research of Bulbus *Fritillariae*. J. Pharmacogn. Phytochem..

[3] Chai S., To K.K., Lin G. (2010). Circumvention of multi-drug resistance of cancer cells by Chinese herbal medicines. Chin. Med..

[4] Yang C.S., Chen G., Wu Q. (2014). Recent scientific studies of a traditional chinese medicine, tea, on prevention of chronic diseases. J. Tradit. Complement. Med..

[5] Namkung W., Thiagarajah J.R., Phuan P., Verkman A.S. (2010). Inhibition of Ca^2+^-activated Cl^−^ Channels by gallotannins as a possible molecular basis for health benefits of red wine and green tea. FASEB J..

[6] Nielsen B., Bermudez I., Bouzat C. (2019). Flavonoids as positive allosteric modulators of α7 nicotinic receptors. Neuropharmacology.

[7] Huang Y., Guo S., Ren S., Chen Y., Zhan Y., An H. (2018). The natural compound cinnamaldehyde is a novel activator of calcium-activated chloride channel. J. Membr. Biol..

[8] Yin Z., Zhang J., Guo Q., Chen L., Zhang W., Kang W. (2019). Pharmacological effects of verticine: Current status. Evid. Based Complement. Altern. Med..

[9] Chen L., Lu X., Liang X., Hong D., Guan Z., Guan Y., Zhu W. (2016). Mechanistic studies of the transport of peimine in the Caco-2 cell model. Acta Pharm. Sin. B.

[10] Xu J., Zhao W., Pan L., Zhang A., Chen Q., Xu K., Lu H., Chen Y. (2016). Peimine, a main active ingredient of Fritillaria, exhibits anti-inflammatory and pain suppression properties at the cellular level. Fitoterapia.

[11] Kan L., Zhao W., Pan L., Xu J., Chen Q., Xu K., Xiao L., Chen Y. (2017). Peimine inhibits hERG potassium channels through the channel inactivation states. Biomed. Pharmacother..

[12] Zhou Y., Ji H., Lin B.-Q., Jiang Y., Li P. (2006). The effects of five alkaloids from bulbus fritillariae on the concentration of cAMP in HEK cells transfected with muscarinic M2 receptor plasmid. Am. J. Chin. Med..

[13] Oh H., Kang D.-G., Lee S., Lee Y., Lee H.-S. (2003). Angiotensin converting enzyme (ACE) inhibitory alkaloids from *Fritillaria ussuriensis*. Planta Med..

[14] Tan H., Zhang G., Yang X., Jing T., Shen D., Wang X. (2019). Peimine inhibits the growth and motility of prostate cancer cells and induces apoptosis by disruption of intracellular calcium homeostasis through Ca 2+/CaMKII/JNK pathway. J. Cell. Biochem..

[15] Hurst R., Rollema H., Bertrand D. (2013). Nicotinic acetylcholine receptors: From basic science to therapeutics. Pharmacol. Ther..

[16] Gotti C., Clementi F. (2004). Neuronal nicotinic receptors: From structure to pathology. Prog. Neurobiol..

[17] Albuquerque E.X., Pereira E.F.R., Alkondon M., Rogers S.W. (2009). Mammalian acetylcholine receptors: From structure to function. Physiol. Rev..

[18] Sine S.M. (2012). End-plate acetylcholine receptor: Structure, mechanism, pharmacology, and disease. Physiol. Rev..

[19] Bouzat C., Mukhtasimova N. (2018). The nicotinic acetylcholine receptor as a molecular machine for neuromuscular transmission. Curr. Opin. Physiol..

[20] Cetin H., Beeson D., Vincent A., Webster R. (2020). The structure, function, and physiology of the fetal and adult acetylcholine receptor in muscle. Front. Mol. Neurosci..

[21] Alberola-Die A., Fernández-Ballester G., González-Ros J.M., Ivorra I., Morales A. (2016). Muscle-type nicotinic receptor blockade by diethylamine, the hydrophilic moiety of lidocaine. Front. Mol. Neurosci..

[22] Katz B., Thesleff S. (1957). A study of the ‘desensitization’ produced by acetylcholine at the motor end-plate. J. Physiol..

[23] Morales A., Aleu J., Ivorra I., Ferragut J.A., Ros J.M.G., Miledi R. (1995). Incorporation of reconstituted acetylcholine receptors from *Torpedo* into the *Xenopus* oocyte membrane. Proc. Natl. Acad. Sci. USA.

[24] Alberola-Die A., Martinez-Pinna J., González-Ros J.M., Ivorra I., Morales A. (2011). Multiple inhibitory actions of lidocaine on *Torpedo* nicotinic acetylcholine receptors transplanted to *Xenopus* oocytes. J. Neurochem..

[25] Alberola-Die A., Fernández-Ballester G., González-Ros J.M., Ivorra I., Morales A. (2016). Muscle-type nicotinic receptor modulation by 2,6-dimethylaniline, a molecule resembling the hydrophobic moiety of lidocaine. Front. Mol. Neurosci..

[26] Cobo R., Nikolaeva M., Alberola-Die A., Fernández-Ballester G., Ros J.M.G., Ivorra I., Morales A. (2018). Mechanisms underlying the strong inhibition of muscle-type nicotinic receptors by tetracaine. Front. Mol. Neurosci..

[27] Heidmann T., Bernhardt J., Neumann E., Changeux J.P. (1983). Rapid kinetics of agonist binding and permeability response analyzed in parallel on acetylcholine receptor rich membranes from *Torpedo marmorata*. Biochemistry.

[28] Cobo R., Nikolaeva-Koleva M., Alberola-Die A., Fernández-Ballester G., Ros J.M.G., Ivorra I., Morales A. (2020). Mechanisms of Blockade of the muscle-type nicotinic receptor by benzocaine, a permanently uncharged local anesthetic. Neuroscience.

[29] Nury H., Poitevin F., Van Renterghem C., Changeux J.-P., Corringer P.-J., Delarue M., Baaden M. (2010). One-microsecond molecular dynamics simulation of channel gating in a nicotinic receptor homologue. Proc. Natl. Acad. Sci. USA.

[30] Wang Z., Cao F., Chen Y., Tang Z., Wang Z. (2018). Simultaneous Determination and pharmacokinetics of peimine and peiminine in *Beagle* dog plasma by UPLC-MS/MS after the oral administration of *Fritillariae ussuriensis* Maxim and *Fritillariae thunbergii* Miq Powder. Molecules.

[31] Chen L., Liu L., Zhu W., Zhang H., Yan Z., Liu H. (2011). Comparative pharmacokinetic studies of peimine and peiminine in rat plasma by LC-MS-MS after oral administration of *Fritillaria thunbergii* Miq. and *Fritillaria thunbergii* Miq.-*Glycyrrhiza uralensis* Fisch. couple extract. Pharmazie.

[32] Unwin N. (2005). Refined structure of the nicotinic acetylcholine receptor at 4Å resolution. J. Mol. Biol..

[33] Unwin N., Fujiyoshi Y. (2012). Gating movement of acetylcholine receptor caught by plunge-freezing. J. Mol. Biol..

[34] Rahman M., Teng J., Worrell B.T., Noviello C.M., Lee M., Karlin A., Stowell M.H., Hibbs R.E. (2020). Structure of the native muscle-type nicotinic receptor and inhibition by snake venom toxins. Neuron.

[35] Unwin N. (2020). Protein–lipid architecture of a cholinergic postsynaptic membrane. IUCrJ.

[36] Ros J.M.G., Llanillo M., Paraschos A., Martinez-Carrion M. (1982). Lipid environment of acetylcholine receptor from *Torpedo californica*. Biochemistry.

[37] Popot J., Demel R.A., Sobel A., Deenen L.L.M., Changeux J. (1978). Interaction of the acetylcholine (nicotinic) receptor protein from *Torpedo marmorata* electric organ with monolayers of pure lipids. Eur. J. Biochem..

[38] Godin J.-R., Roy P., Quadri M., Bagdas D., Toma W., Narendrula-Kotha R., Kishta O.A., Damaj M.I., Horenstein N.A., Papke R.L. (2020). A silent agonist of α7 nicotinic acetylcholine receptors modulates inflammation ex vivo and attenuates EAE. Brain Behav. Immun..

[39] Alberola-Die A., Reboreda A., Lamas J.A., Morales A. (2013). Lidocaine effects on acetylcholine-elicited currents from mouse superior cervical ganglion neurons. Neurosci. Res..

[40] Iwashina T. (2003). Flavonoid function and activity to plants and other organisms. Biol. Sci. Space.

[41] Changeux J.-P., Taly A. (2008). Nicotinic receptors, allosteric proteins and medicine. Trends Mol. Med..

[42] Ivorra I., Ndez A.F., Gal B., Aleu J., Lez-Ros J.G., Ferragut J., Morales A. (2002). Protein orientation affects the efficiency of functional protein transplantation into the *Xenopus* Oocyte Membrane. J. Membr. Biol..

[43] Kusano K., Miledi R., Stinnakre J. (1982). Cholinergic and catecholaminergic receptors in the *Xenopus* oocyte membrane. J. Physiol..

[44] Olivera-Bravo S., Ivorra I., Morales A. (2005). The acetylcholinesterase inhibitor BW284c51 is a potent blocker of *Torpedo nicotinic* AchRs incorporated into the *Xenopus* oocyte membrane. Br. J. Pharmacol..

[45] Olivera-Bravo S., Ivorra I., Morales A. (2007). Diverse inhibitory actions of quaternary ammonium cholinesterase inhibitors on *Torpedo nicotinic* ACh receptors transplanted to *Xenopus* oocytes. Br. J. Pharmacol..

[46] Encinar J.A., Fernández-Ballester G.J., Galiano-Ibarra V., Micol-Molina V. (2015). In silico approach for the discovery of new PPARγ modulators among plant-derived polyphenols. Drug Des. Dev. Ther..

[47] Krieger E., Nielsen J.E., Spronk C.A., Vriend G. (2006). Fast empirical pKa prediction by Ewald summation. J. Mol. Graph. Model..

[48] Verdura S., Cuyàs E., Cortada E., Brunet J., Lopez-Bonet E., Martin-Castillo B., Bosch-Barrera J., Encinar J.A., Menendez J.A. (2020). Resveratrol targets PD-L1 glycosylation and dimerization to enhance antitumor T-cell immunity. Aging.

[49] Encinar J.A., Menendez J.A. (2020). Potential drugs targeting early innate immune evasion of SARS-Coronavirus 2 via 2’-O-methylation of viral RNA. Viruses.

[50] Voss N.R., Gerstein M. (2010). 3V: Cavity, channel and cleft volume calculator and extractor. Nucleic Acids Res..

[51] Pettersen E.F., Goddard T.D., Huang C.C., Couch G.S., Greenblatt D.M., Meng E.C., Ferrin T. (2004). UCSF Chimera? A visualization system for exploratory research and analysis. J. Comput. Chem..

