# Supplementary material for: Peimine, an Anti-Inflammatory Compound from Chinese Herbal Extracts, Modulates Muscle-Type Nicotinic Receptors
Source: Int J Mol Sci. 2021 Oct 19;22(20):11287. doi: 10.3390/ijms222011287 (PMC8539251; doi:10.3390/ijms222011287)
Supplement: Supplementary file 1 [file ijms-22-11287-s001.zip › ijms-1418114-supplementary.pdf]

International Journal of *Molecular Sciences*

MDPI

SUPPLEMENTARY MATERIAL

Peimine, an Anti-Inflammatory Compound from Chinese Herbal Extracts, Modulates Muscle-Type Nicotinic Receptors

Armando Alberola-Die [1], José Antonio Encinar [2], Raúl Cobo [1], Gregorio Fernández-Ballester [2], José Manuel González-Ros [2], Isabel Ivorra [1] and Andrés Morales [1,*]

[1] División de Fisiología, Departamento de Fisiología, Genética y Microbiología, Universidad de Alicante, Apdo. 99, E-03080 Alicante, Spain; alberoladie.armando@ua.es (A.A.-D.); raulcobo22@gmail.com (R.C.); isabel.ivorra@ua.es (I.I.)

[2] Instituto de Investigación, Desarrollo e Innovación en Biotecnología Sanitaria de Elche (IDiBE), Universidad Miguel Hernández, E-03202 Elche, Spain; jant.encinar@umh.es (J.A.E.); gregorio@umh.es (G.F.-B.); gonzalez.ros@umh.es (J.M.G.-R.)

* Correspondence: andres.morales@ua.es; Tel.: +34-96-590-3949

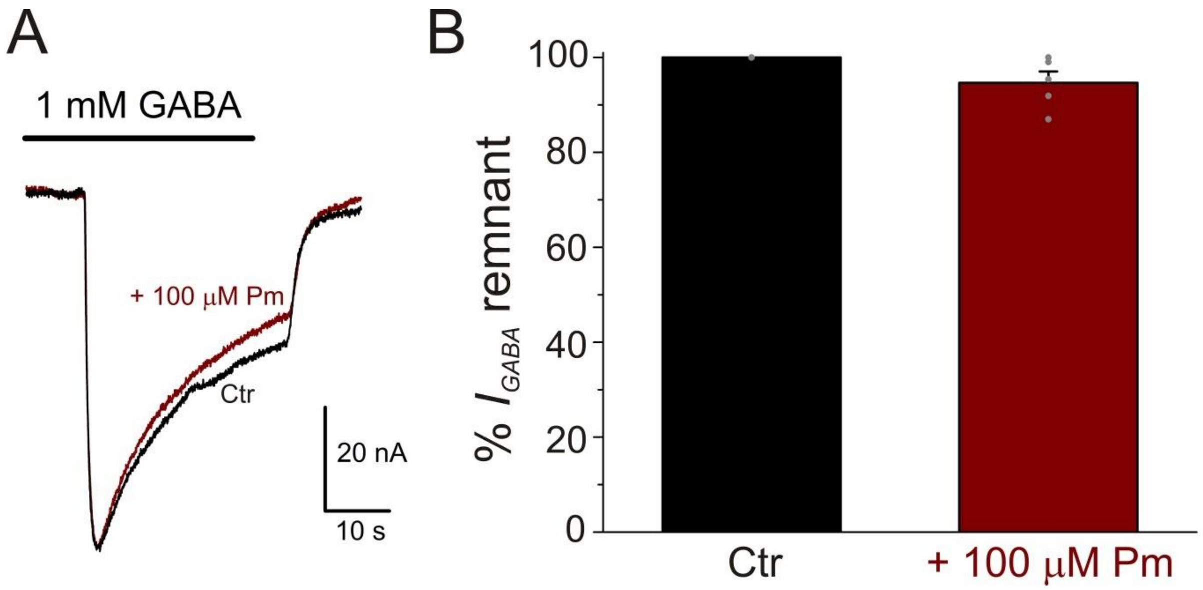

Supplementary Figure S1. Lack of effect of Pm on GABA-elicited currents (I_{GABA}). **A**. Superimposed I_{GABA}s elicited by 1 mM GABA alone (black recording) or together with 100 μM Pm (red trace) in an oocyte previously injected with synaptosomal-enriched rat-brain membranes. Notice that Pm, even at concentrations as high as 100 μM, did not attenuate I_{GABA}. **B**. Column graph showing the percentage of I_{GABA} in the control (black) and Pm (red) groups. There was not significant differences between both groups ($p < 0.05$, *t*-test). Data are from 5 oocytes of 3 donor frogs.

Scheme 1. Putative nAChR residues interacting with Pm in the open and theclosed conformation, as predicted from molecular docking simulations.

nAChR State	Dmain	Pm Cluter	Binding energy (Kcal/mo)	**Residues** (subunit name-residue name-number)
**OPEN**	**TMD**	1	-10.12	β (A258, V261, F262, L265), γ (L265, A266, V269, F270, L273), αγ (V255,F256, V259, E262, I263), δ (F266, L267, Q270)
		2	-10.57	αδ (T244, L245, I247, S248), β (S250, I253, S254, L257, A258, V261, F262),<br>γ (C262, L265, A266, V269, F270), αγ (S248, L251, S252, V255), δ (T252,S256, L259)
		3	-11.24	αγ (Y213, F214, N217, V218, I220, P221, I260, L263, I264, T267), δ (F266,A269, Q270, P273, L277, L281, L286, M290)
		4	-11.06	αδ (F135, F137, I210, F214, V215, V218, I219, C222, I260, I264, Y277, F280, T281, F284, V285, A427, G428, I431, E432)
		5	-10.82	δ (F134, L219, Y221, I222, I225, I226, C229, V230, V272, S276, Y285, L286, F288, V289, V292, A464, I465, T468)
		6	-10.71	β (E45, F135, Q185, W186, R215, P217, F219, Y220, I221, T224, V270, T273, S274, V277, P278, I279, I280, I281, Y283, L284), γ (L283)
		7	-10.68	γ (E47, F137, F139, W141, R223, K224, P225, Y228, V229, F232, I233, L278, T281, V285, L287, Y291, F294, I295, F471, F478)
		8	-10.56	αδ (I219, C222, L223, F225, S226, T229, V230, F233, F284, V285, S287, S288, V291, I417, I420, G421, S424, V425, G428)
		9	-10.42	γ (F137, R223, P225, Y228, V229, F232, I233, C236, L278, T281, V285,L287, Y291, L292, F294, I295, F471)
		10	-10.29	αγ (F135, F137, F214, V218, I219, C222, I264, L273, Y277, M278, F280, T281, F284, S424, V425, G428, R429, I431)
		11	-10	β (Y220, I221, Y223, T224, P227, L266, K269, V270, E272, T273), γ (L273,Q276, P279, E280, L283)
		12	-9.87	αγ (E175, P211, Y213, F214, N217, L263, S266, T267), δ (F266, A269, Q270, K271, P273, E274, L277, L281, M290)
		13	-9.3	γ (E186, N187, G188, E189, W190, P225, L226, F227), αγ (V46, N47, Q48,Y127, I130, V132, S269, P272)
	**ECD**	14	-11.97	γ (S40, N55, W57, P123), αγ (V91, Y93, N94, A96, W149, T150, Y151,D152, P197, Y198)
		15	-10.89	αδ (V91, L92, Y93, A96, I148, W149, T150, Y190, C192, C193, Y198), δ (T35, T37, N52, W54, E56, Y116, L118, P120)
		16	-10.72	αδ (I148, W149, T150, Y151, D152, Y190, P197, Y198), δ (T35, W54, R78,L108, Y116, L118, E175)
		17	-10.49	αδ (L87, P88, D89, L90, V91, A96, G98, D99, F100, V103, H104, M105, W118, W149), δ (V101, Y104, P120)
**CLOSED**	**TMD**	1	-11.2	αδ (T244, L245, I247, S248, L251), β (I253, S254, L257, A258, V261, F262),<br>γ (C262, L265, A266, V269, F270), αγ (S248, L251, S252, V255), δ (T252,S256, L259, I263)
		2	-12.87	β (Q185, L218, F219, Y220, V222, Y223), γ (T48, V134, A282, L283, A284,V285, P286, L287, I288, G289, L292, M293, M296)
		3	-11.49	β (F135, Q185, P217, F219, Y220, I221, T224, K269, V270, T273, S274, V277, P278, I279, I280, I281, Y283, L284, I287), γ (L283)
		4	-10.87	δ (F134, F136, K217, L219, Y221, I222, I225, I226, V272, P273, S276, L281,I282, Y285, L286, V289, I465, T468)
		5	-10.84	αγ (F135, F137, I210, F214, V215, V218, I219, C222, I264, L273, Y277, M278, F280, T281, F284, S424, V425, G428, R429)
		6	10.75	αγ (F135, F137, I210, F214, V215, V218, I219, L273, Y277, M278, F280, T281, F284, G421, S424, V425, G428, R429)
		7	-10.18	β (P242, P243, D244, A245, G246, E247, M249, L304, H305, H306, R307,S308, T311, H312, Y430, V431, A432, A435, D436), γ (P317, S318)
		8	-10.17	αδ (F137, I210, F214, V215, V218, I219, C222, L223, F225, S226, F280, F284, G421, T422, S424, V425, G428)
		9	-10.11	αδ (F137, I210, F214, V215, V218, I219, C222, L223, F225, Y277, F280, T281, F284, G421, T422, S424, V425)
		10	-9.61	αγ (E172, S173, G174, E175, P211, Y213, V271), δ (K45, E46, A130, V131,S276, L277, N278, V279, P280, L281)
	**ECD**	11	-11.52	αδ (L87, P88, D89, L90, V91, L92, A96, D97, G98, D99, F100, V103, H104,W118, F124, W149), δ (V101, P120)
		12	-10.98	γ (S40, N55, W57, V104, P123), αγ (V91, Y93, I148, W149, T150, Y151,D152, P197, Y198)
		13	-10.6	αδ (V91, L92, Y93, N94, I148, W149, T150, Y190, Y198), δ (T35, T37, W54,E56, Y116, L118, P120)
		14	-10.39	γ (W57, V104, P123, I125), αγ (D89, L90, V91, L92, Y93, N94, N95, A96,D99, F100, I148, W149, Y198)

15	-10.34	γ (S40, N41, N55, W57, P123), αγ (V91, L92, Y93, N94, N95, A96, G147,I148, W149, T150, Y190, P197, Y198)
16	-9.59	αγ (E2, E4, L7, V8, L11, A70, D71, G74, I75, I78, R79, L80, P81), δ (R17,L24, Y150)
